# Colorectal cancer-derived extracellular vesicles induce transformation of fibroblasts into colon carcinoma cells

**DOI:** 10.1186/s13046-019-1248-2

**Published:** 2019-06-14

**Authors:** Mohamed Abdouh, Matteo Floris, Zu-Hua Gao, Vincenzo Arena, Manuel Arena, Goffredo Orazio Arena

**Affiliations:** 1 Cancer Research Program, McGill University Health Centre-Research Institute, 1001 Decarie Boulevard, Montreal, Quebec H4A 3J1 Canada; 2 Department of Biomedical Sciences, Sassari University, Piazza Universita 11, Sassari, Italy; 3 Department of Pathology, McGill University Health Centre-Research Institute, 1001 Decarie Boulevard, Montreal, Quebec H4A 3J1 Canada; 4 Department of Obstetrics and Gynecology, Santo Bambino Hospital, via Torre del Vescovo 4, Catania, Italy; 5 Department of Surgical Sciences, Organ Transplantation and Advances Technologies, University of Catania, via Santa Sofia 84, Catania, Italy; 6 Department of Surgery, McGill University, St. Mary Hospital, 3830 Lacombe Avenue, Montreal, Quebec H3T 1M5 Canada

**Keywords:** Extracellular vesicles, Colorectal Cancer, Metastasis, Genetic material, Horizontal transfer

## Abstract

**Background:**

We reported that horizontal transfer of malignant traits to target cells is a potential pathway to explain cancer dissemination. Although these results were encouraging, they were never corroborated by data showing the molecular mechanisms responsible for the observed phenomenon.

**Methods:**

In the present study, we exposed *BRCA1*-KO fibroblasts to extracellular vesicles (EVs) isolated from a colon cancer cell line (HT29) and from sera of patients with colorectal cancer. Three weeks after exposure, fibroblasts were injected subcutaneously into NOD-SCID mice. Whole genome sequencing, transcriptome analysis and RNA sequencing of cancer EVs and fibroblasts prior and after exposure to cancer EVs were performed.

**Results:**

Phenotypical transformation of the fibroblasts into colon cancer cells was confirmed by histopathological study of the xenotransplants. We observed that EV-mediated transfer of cancer microRNAs was responsible for the transition from a mesenchymal to an epithelial phenotype (MET) in the treated fibroblasts as well as activation of cell cycle progression and cell survival pathways. DNA and RNA sequencing suggested that cancer DNA was transferred and possibly transcribed in target cells. Furthermore, injection of colon cancer EVs in the tail vein of NOD-SCID mice determined neoplastic transformation and metastases in the lungs of the mice confirming for the first time the hypothesis that transfer of malignant epithelial cancer traits to distant target cells is a concept applicable to in vivo models.

**Conclusions:**

These discoveries shed new light into the molecular mechanisms behind the horizontal transfer of malignant traits and confirm the notion that metastatic disease might be reproduced through transfer of circulating genetic material.

**Electronic supplementary material:**

The online version of this article (10.1186/s13046-019-1248-2) contains supplementary material, which is available to authorized users.

## Background

Metastatic disease is the leading cause of cancer related mortality [1]. It is considered to be a complex multistep biological process controlled by distinct genes and signaling pathways [2]. According to the “seed to soil” hypothesis, the metastatic process initiated by the detachment of cells is followed by invasion into the neighboring tissues, penetration trough the basement membrane, dissemination via the blood flow and engraftment of the malignant cells in distant sites from the primary tumor [2–4]. In the past decade, it has been proposed that changes in cell phenotype between the epithelial and mesenchymal states, defined as the epithelial–mesenchymal transition (EMT) and mesenchymal–epithelial transition (MET), could be critical events responsible for the metastatic cascade [2]. During these events, epithelial cells would acquire fibroblast like properties with the expression of the mesenchymal marker vimentin [5] and would exhibit reduced cell-cell adhesion and increased motility via EMT, which eventually would facilitate the escape of tumor cells from primary tumors [2]. Subsequently, the cells would undergo the reverse transition, which would allow the reacquisition of their epithelial state (MET) and would enable the malignant cells to escape the circulation and home to the target organs. The legitimacy of this model has been strengthened by the accumulating evidence that down-regulation of the expression of the epithelial marker E-cadherin (CDH1) is a crucial step in the progression from dysplasia to invasive carcinoma [6]. Three groups of transcriptional regulators able to suppress CDH1 expression and also involved in the metastatic process have been identified and studied extensively: the transcription factors of the Snail zinc-finger family, including SNAI1 and SNAI2, the zinc-finger E-box-binding homeobox family proteins ZEB1and ZEB2 and the basic helix loop helix family of transcription factors, including TWIST1, TWIST2, and E12/E47 [7].

Lately, the initial enthusiasm has been curbed by the lack of consistency of data supporting the notion that the same cell is able to revert the process (MET) and therefore downregulate the expression of vimentin, upregulate the expression of CDH1 with subsequent loss of migratory freedom, adoption of apico-basal polarization and expression of the junctional complexes that are hallmarks of epithelial tissues [8, 9]. Furthermore, increasing evidence of significant discrepancies between the genotype of the primary cancer cell and its metastatic counterpart [10, 11], coupled with conflicting explanations on the phenomenon of late metastases, have raised more doubts on the true identity of metastatic cells.

Garcia-Olmo in the late nineties [12, 13] and more recently Trejo-Becerril [14] and our group [15–17] have brought forward the hypothesis that the metastatic process might not be solely due to primary tumor cells spreading to distant metastatic sites but might occur via transfer of biologically active cancer circulating factors to susceptible target cells located in distant organs. Our group suggested and demonstrated in vitro that circulating cancer exosomes induce malignant transformation of target cells even at distance through horizontal transfer of malignant traits [15]. These extracellular vesicles (EVs) are released from a plethora of cell types including cancer cells and are isolated from a variety of body fluids as well as serum [18, 19]. Interestingly, the amount of EVs recovered increases with advancing stage of malignancies, pointing to potential roles in cancer progression and invasion [17, 19, 20]. Specifically, cancer EVs carry in their lumen genetic materials and proteins that favor tumor growth and alter the tumor niche micro-environment [15–17, 20].

Our experiments confirmed that different types of oncosuppressor mutated cell lines are able to turn malignant when exposed to either EVs collected from cancer cells’ conditioned media or EVs extracted from cancer patients’ sera. Notably the exposed cells displayed features resembling those of the primary tumors with gain of immunohistochemical features similar or identical to the cancer cells that release the EVs [15].

In the present study, for the first time, we shed light on the molecular mechanisms behind this phenomenon. We profiled DNA, mRNA and miRNA expression in colorectal cancer-derived EVs and target fibroblasts prior and following exposure to the cancer EVs. We confirmed that the phenotypical transformation of the fibroblasts is associated with cancer EVs DNA transfer and we showed data that suggest that EVs DNA is actively transcribed in the fibroblasts after the exposure. We further demonstrated that a definite set of miRNA families, transferred from the colon cancer derived EVs to the fibroblasts, activate cell cycle progression and cell survival pathways. Remarkably, we noted that the uptake of cancer miRNAs was concomitant to downregulation of transcription factors that prompted a mesenchymal to epithelial transition of the fibroblasts that acquired epithelial features and immunohistochemistry markers, suggestive of complete colorectal cancer differentiation. Furthermore, we showed that injection of cancer EVs into the blood stream of NOD/SCID mice can prompt malignant transformation at distance, confirming for the first time that horizontal transfer of epithelial malignant traits through EVs might also be applied to in vivo systems.

## Methods

### Patients’ recruitment and characteristics of cancers

Patients for the current study were recruited from the department of General Surgery at the Royal Victoria Hospital and St-Mary’s Hospital (Montreal, Canada). They underwent an informed consent for blood collection in accordance to a protocol approved by the Ethics Committee of our institution (SDR-10-057). Blood samples were collected and categorized in two cohorts: healthy individuals (13 control patients; CTL1-CTL13) and patients with colorectal cancer (18 patients; Case1-Case18) (Table 1). The inclusion criteria to be enrolled in the healthy cohort were: (i) age (30–60 year old), (ii) no signs and symptoms or personal history of cancer and (iii) family history negative for cancer.

**Table 1:** Exposure conditions and clinical features of patients recruited for this study

Medium	Age (y)	Gender	Disease	Mean^a^	SD^a^
Control Medium	–	–	–	0	0
Control Medium	–	–	–	0	0
Control Medium	–	–	–	0	0
Control Medium	–	–	–	0	0
Control Medium	–	–	–	0	0
Exo-CM-HT29	–	–	Colon Cancer Cell Line	0,367	0,039
Exo-CM-HT29	–	–	Colon Cancer Cell Line	0,373	0,048
Exo-CM-HT29	–	–	Colon Cancer Cell Line	0,359	0,019
Exo-CM-HT29	–	–	Colon Cancer Cell Line	0,369	0,016
Exo-CM-HT29	–	–	Colon Cancer Cell Line	0,276	0,025
Control serum 1	30	Male	Healthy	0	0
Control serum 2	35	Female	Healthy	0	0
Control serum 3	42	Female	Healthy	0	0
Control serum 4	41	Male	Healthy	0	0
Control serum 5	37	Male	Healthy	0	0
Control serum 6	30	Female	Healthy	0	0
Control serum 7	33	Female	Healthy	0	0
Control serum 8	35	Male	Healthy	0	0
Control serum 9	39	Female	Healthy	0	0
Control serum 10	38	Male	Healthy	0	0
Control serum 11	45	Male	Healthy	Scratch assay	
Control serum 12	39	Female	Healthy	Scratch assay	
Control serum 13	28	Male	Healthy	Scratch assay	
Exo-Case1	63	Male	Colorectal Cancer	0,509	0,015
Exo-Case2	78	Female	Colorectal Cancer	0,708	0,071
Exo-Case3	65	Male	Colorectal Cancer	0,838	0,074
Exo-Case4	72	Female	Colorectal Cancer	1564	0,256
Exo-Case5	64	Female	Colorectal Cancer	1038	0,162
Exo-Case6	59	Male	Colorectal Cancer	0,200	0,023
Exo-Case7	73	Male	Colorectal Cancer	1440	0,169
Exo-Case8	43	Male	Colorectal Cancer	1331	0,171
Exo-Case9	66	Female	Colorectal Cancer	0,485	0,052
Exo-Case10	63	Female	Colorectal Cancer	0,437	0,033
Exo-Case11	49	Female	Colorectal Cancer	0,341	0,062
Exo-Case12	45	Male	Colorectal Cancer	0,361	0,037
Exo-Case13	66	Male	Colorectal Cancer	1903	0,308
Exo-Case14	66	Male	Colorectal Cancer	Scratch assay	
Exo-Case15	61	Female	Colorectal Cancer	Scratch assay	
Exo-Case16	46	Female	Colorectal Cancer	Scratch assay	
Exo-Case17	49	Male	Colorectal Cancer	Scratch assay	
Exo-Case18	56	Male	Colorectal Cancer	Scratch assay	

^a^ Size of xenotransplants. BRCA1-KO fibroblasts were treated with different media for 3 weeks. Treated cells were injected subcutaneously into NOD/SCID mice that were followed for 4 weeks for tumors growth. Xenotransplants were measured and data were expressed as mean +/− SD (*n* = 2–3 mice). *P* value = 0.0006 when comparing control medium-exposed cells (*n* = 5) to HT29 cells conditioned medium EVs-exposed cells (*n* = 5). *P* value = 0.0074 when comparing healthy serum EVs-treatd cells (*n* = 10) to cancer patients serum EVs-treated cells (*n* = 13)

Exo-CM-HT29 and Exo-Case: EVs isolated respectively from HT29 cells conditioned medium and cancer patient’s sera. Conditioned medium was collected from HT29 cells at 80–90% confluence. EVs were isolated by differential ultracentrifugation

### Blood collection and serum preparation

Blood samples were collected from a peripheral vein in vacutainer tubes (Becton Dickinson) containing clot-activation additive and a barrier gel to isolate serum. Blood samples were incubated for 60 min at room temperature to allow clotting and subsequently were centrifuged at 1500 g for 15 min. Serum was collected and a second centrifugation was performed on the serum at 2000 g for 10 min, to clear it from any contaminating cells. Serum samples were aliquoted and stored at − 80 °C until use.

### Cell lines and culture conditions

Human colorectal adenocarcinoma HT-29 cell line (ATCC) was used to collect conditioned medium for EVs isolation. We used human *BRCA1*-deficient fibroblasts, previously described [16], as target cells for exposure to EVs. Human colorectal adenocarcinoma Colo320 cell line (ATCC) was used as positive control for the validation of the antibodies used in this study. Murine colon adenocarcinoma MC38 cell line was given by Dr. Pnina Brodt. Cells were maintained in DMEM-F12 medium supplemented with 10% fetal bovine serum, and penicillin/streptomycin antibiotics (Wisent, Saint-Bruno, Canada). When *BRCA1*-KO fibroblasts reached 30% confluence, they were treated with complete DMEM-F12 medium supplemented with 30 μg/ml of EVs isolated from HT29 cells conditioned medium, cancer patient sera or control sera (as described below). Cells were maintained in these media at 37 °C in humidified atmosphere containing 95% air and 5% CO_2_ with medium change every second day for 3 weeks. When cells reached 80–90% confluence, they were passaged 1 in 6 using 0.05% Trypsin-EDTA (Wisent, Saint-Bruno, Canada).

### Extracellular vesicles isolation and labeling

For conditioned medium recovery, HT29 cells were cultured in DMEM-F12 medium supplemented with 10% EV-free fetal bovine serum. Supernatant was collected when cells reached 80–90% confluence. Human serum diluted in PBS and HT29 cells conditioned medium were centrifuged at 300 g for 10 min to remove contaminating cells. This was followed by serial centrifugation at 1200 g for 20 min, and 10,000 g for 30 min to remove debris and large vesicles. After supernatant passage through a 0.2 μm filter, EVs were pelleted by ultracentrifugation (Beckman ultracentrifuge, Beckman Coulter) at 100,000 g for 70 min. The pelleted EVs were washed twice in Hank’s Balanced Salt Solution (HBSS), re-suspended in HBSS, and stored at − 80 °C until used.

For EVs uptake analysis, aliquots of EVs were labeled using the PKH26 dye following the manufacturer recommendations (Sigma, Oakville, Canada). Labeled EVs were pelleted by ultra-centrifugation for 70 min at 100,000 x g at 4 °C. The pellet was washed in HBSS with an ultra-centrifugation using the same parameters. The pelleted EVs were re-suspended in HBSS and added to cells in 8-well chamber slides (VWR, Mont-Royal, Canada) for 3 h. Cells were washed, fixed for 10 min with paraformaldehyde 4%. Slides were mounted with coverslip in VECTASHIELD Mounting Medium with DAPI (Vector Laboratories, Burlington, Canada). Stained cells were visualized using an LSM780 confocal microscope (Zeiss, Toronto, Canada).

For EVs uptake inhibition, fibroblasts were treated for 1 h. with different receptor antagonists: Anti-β4 Integrin (10 μg/ml, ASC-8, ab77801, abcam), Cytostatin (1.4 μg/ml, 19,602, Cedarlane), and Heparin (10 μg/ml, H3149, Sigma). In parallel, EVs were treated for 2 h. with RGD (300 nM, 14,501–1, Cedarlane), and Collagenase I (500 μg/ml, C0130, Sigma). Afterwards, cells were washed, mixed with treated EVs and incubated for 3 h. EVs internalization was analyzed by flow cytometry. Cells were acquired using a FACSCalibur flow cytometer (Becton-Dickinson) at a flow rate of ~ 300 cells/second. Dead cells and cell debris were excluded from acquisition by gating intact cells on a Forward Scatter and Side Scatter biparametric plot.

For EVs DNA transfer into target cells analysis, EVs were labeled with acridine orange (AO; 20 μM; Invitrogen) for 90 min to tag DNA. Labeled EVs were recovered by ultracentrifugation in HBSS and added to cells in 8-well chamber slides for 24 h. Cells were washed and fixed for 10 min with paraformaldehyde 4%. Slides were mounted with coverslip in Mounting Medium with DAPI. Cells were visualized using an LSM780 confocal microscope.

For EVs RNA transfer into target cells analysis, HT29 cells (EVs donor cells) were transfected with the BrUTP (480 μM; Sigma) using Lipofectamine 3000 (Invitrogen) following manufacturer recommendations. 24 h later conditioned medium was collected for the isolation of EVs. *BRCA1*-KO fibroblasts were exposed to these EVs for 12 h. Cells were analyzed for RNA-BrUTP transfer by flow cytometry using anti-BrdU-FITC antibody (Pharmingen).

### Extracellular vesicles characterization

Morphological examination of isolated EVs was done using transmission electron microscope (FEI Titan Krios 300 kV Cryo-STEM). Briefly, 20 μl of EVs preparation were loaded on a copper grid and stained with 2% phosphotungstic acid. Samples were dried by incubating them for 10 min under an electric incandescent lamp. In Parallel, an aliquot of EVs samples was run on a Nanosight NS500 system (Nanosight Ltd., Amesbury, UK), and size distribution was analyzed using the NTA 1.3 software.

### Population doubling level (PDL) calculation

Cells were considered at population doubling zero at the first time they were exposed to control and cancer EVs. At every passage, cell number was determined and population doubling was calculated using the following formula; PDL = log(Nh/Ni)/log2, where Nh is the number of cells harvested at the end of the incubation time and Ni is the number of cells inoculated at the beginning of the incubation time. Cumulative PDL was calculated by adding the previous calculated PDL.

### Cell viability assessment

For the analysis of apoptosis, we used AnnexinV/7-AAD labeling. Briefly, dissociated cells were resuspended in AnnexinV binding buffer (BD Biosciences), and stained with FITC-AnnexinV (PharMingen). Just before cell acquisition, 5 μl of 7-AAD was added and cells were acquired in a FACS Calibur flow cytometer (Becton-Dickinson).

### Protein preparation and mass spectrometry

Plasma membranes proteins were enriched from fibroblast lysates (Fibroblasts control vs. *BRCA1*-KO fibroblasts) using the Plasma Membrane Protein Extraction Kit (Abcam, MA, USA). Proteins were also prepared from pelleted EVs. Protein samples were run on a stacking gel, and gel bands were reduced with DTT, alkylated with iodoacetic acid and then digested with trypsin with re-solubilization in 0.1% aqueous formic acid/2% acetonitrile. Peptides were loaded onto a Thermo Acclaim Pepmap precolumn (Thermo, 75uM ID X 2 cm C18 3uM beads), and onto an Acclaim Pepmap Easyspray analytical column separation (Thermo, 75uM X 15 cm with C18 2uM beads) using a Dionex Ultimate 3000 uHPLC at 220 nl/min with a gradient of 2–35% organic (0.1% formic acid in acetonitrile) over 4 h. Peptides were analyzed using a Thermo Orbitrap Fusion mass spectrometer operating at 120,000 resolution (FWHM in MS1, 15,000 for MS/MS) with HCD sequencing all peptides with a charge of 2+ or greater. The raw data were converted into MGF format (Mascot Generic Format) searched using Mascot 2.3 against human sequences (Swissprot). The database search results were loaded onto Scaffold Q+ Scaffold_4.7.2 (Proteome Sciences) for spectral counting, statistical treatment and data visualization.

### Whole genome sequencing and RNA analyses

Aliquots of EVs resuspended in HBSS were treated either with 0.2 units/μl of Shrimp DNase (Affymetrix) for 30 min at 25 °C to digest extra-vesicular DNA or 50 μg/ml of RNaseA (Fermentas) for 15 min at 37 °C to digest extra-vesicular RNA. Digestion reaction was stopped by heat inactivation at 70 °C for 5 min. Treated EVs were recovered by ultracentrifugation as stated before. EVs pellet was suspended in HBSS and added to *BRCA1*-KO fibroblasts and cultures were maintained for 6 weeks with medium refreshment every second day.

For DNA transfer analysis, genomic and EVs DNA were extracted using the GenElute Mammalian Genomic DNA Kit (Sigma) and the QIAamp DNA kit (Qiagen), respectively. DNA was eluted in 50 μl of 10 mM Tris pH 8.0 and submitted to the McGill University and Génome Québec Innovation Centre for DNA quality control, shotgun library preparation and Illumina HiSeq DNA sequencing. Briefly, DNA was quantified using the Quant-iT™ PicoGreen® dsDNA Assay Kit (Life Technologies) and its integrity assessed on agarose gels. Libraries were generated using the Lucigen NxSeq AmpFREE Low DNA Library Kit (Lucigen) according to the manufacturer’s recommendations (1000 ng input). Libraries were quantified using the Quant-iT™ PicoGreen® dsDNA Assay Kit (Life Technologies) and the Kapa Illumina GA with Revised Primers-SYBR Fast Universal kit (Kapa Biosystems). Average size fragment was determined using a LabChip GX (PerkinElmer) instrument. The DNA libraries were normalized and then denatured in 0.05 N NaOH and neutralized using HT1 buffer. ExAMP was added to the mix following the manufacturer’s instructions. The DNA pool was loaded at 200 pM on a Illumina cBot and the flowcell was ran on a HiSeq X for 2 × 151 cycles (paired-end mode). A phiX library was used as a control and mixed with libraries at 1% level. The Illumina control software was HCS HD 3.4.0.38, the real-time analysis program was RTA v. 2.7.7. Program bcl2fastq2 v2.18 was then used to demultiplex samples and generate fastq reads.

Whole genome sequencing data was analyzed for variants using GenPipes pipeline. This pipeline follows the stepwise procedures of the well-known BROAD Institute GATK best practices. Raw reads derived from the sequencing instrument were quality trimmed and adapter clipped using Trimmomatic to obtain a high-quality set of reads for sequence alignment (sam/bam) file generation. The trimmed reads were aligned to a reference genome (b37) using a fast, memory-efficient Burrows-Wheeler transform (BWT) aligner BWA-mem. Mapped reads were further refined using GATK and Picard program suites to improve mapping near insertions and deletions (indels; GATK indel realigner), remove duplicate reads with same paired start site (Picard mark duplicates) and improve quality scores (GATK base recalibration). Variants were called using GATK haplotype caller in GVCF mode to allow efficient downstream merging of multiple samples into one variant file to streamline downstream variant processing procedures which include normalization and decomposition of multi nucleotide polymorphisms (MNPs), functional annotation with SNPeff and variant annotations using the Gemini framework which provides quality metric and extensive metadata to help further prioritize variants. Data were visualized using the Integrative Genomics Viewer (IGV) program.

For RNA transcriptome analysis, total cellular and EVs RNA were isolated using the TRIzol reagent (Invitrogen). RNA preparations were cleanup using the RNeasy MinElute Cleanup kit (Qiagen). 2 μg of each cellular and EVs RNA samples were submitted to the McGill University and Génome Québec Innovation Centre for RNA quality control and concentration determination using the Agilent 2100 Bioanalyzer (Thermo Scientific). RNA was converted to cDNA, transcribed and labeled, and then hybridized to human Clariom D Array (Affymetrix). The chip was scanned according to the standard protocol recommended by Affymetrix. The miRNA array profiling was performed using the Affymetrix GeneChip miRNA Array 1.0. Data were analysed using the Transcriptome Analysis Console.

For RNA sequencing, RNA samples were used as templates for library construction, and subsequent sequencing using the Illumina HiSeq4000 PE100 sequencing lane following manufacturer’s instructions. Briefly, libraries were generated from 250 ng of total RNA as following: mRNA enrichment was performed using the NEBNext Poly(A) Magnetic Isolation Module (New England BioLabs). cDNA synthesis was achieved with the NEBNext RNA First Strand Synthesis and NEBNext Ultra Directional RNA Second Strand Synthesis Modules (New England BioLabs). The remaining steps of library preparation were done using and the NEBNext Ultra II DNA Library Prep Kit for Illumina (New England BioLabs). Adapters and PCR primers were purchased from New England BioLabs. Libraries were quantified using the Quant-iT™ PicoGreen® dsDNA Assay Kit (Life Technologies) and the Kapa Illumina GA with Revised Primers-SYBR Fast Universal kit (Kapa Biosystems). Average size fragment was determined using a LabChip GX (PerkinElmer) instrument. The RNA libraries were normalized and then denatured in 0.05 N NaOH and neutralized using HT1 buffer. ExAMP was added to the mix following the manufacturer’s instructions. The RNA pools were loaded at 200pM on an Illumina cBot and the flowcell was ran on a HiSeq 4000 for 2 × 100 cycles. A phiX library was used as a control and mixed with libraries at 1% level. The Illumina control software was HCS HD 3.4.0.38, the real-time analysis program was RTA v. 2.7.7. Program bcl2fastq2 v2.18 was then used to demultiplex samples and generate fastq reads.

The analysis of RNA-Seq reads was performed by following GATK Best Practices workflow for SNP and indel calling on RNAseq data available on the GATK Best Practices website [https://gatkforums.broadinstitute.org/gatk/discussion/3891/calling-variants-in-rnaseq]. In brief, main differences with the DNA-Seq pipeline are the following: i) for the alignment step, we used STAR aligner instead of the BWA aligner; ii) a tool SplitNCigarReads, specially developed for RNAseq, has been used to splits reads into exon segments and hard-clip any sequences overhanging into the intronic regions; iii) the GATK HaplotypeCaller was used for variant calling with the options *dontUseSoftClippedBases* and *recoverDanglingHeads*.

The variant calling procedure was also performed with the Opossum [21] and Platypus [22] tools (with following filters: Total number of reads at that position: ≥ 10, only QUAL filter passing and remove indels).

### Quantitative real-time PCR

All primers were designed to flank individual exons and tested by PCR of RT^+^ and RT^−^ control extracts. 1 μg of cellular or 100 ng of EVs RNA were reverse-transcribed (RT) using the MML-V reverse transcriptase (Invitrogen). Quantitative real-time PCR (qPCR) was performed using the Platinum SYBR Green SuperMix (Invitrogen) and an ABI Prism 7500 Real-Time PCR apparatus (Applied Biosystems). Primer sets used were as shown in Additional file 1: Table S1. GAPDH was used as an internal standard for data calibration. The 2^-ΔΔCt^ formula was used for the calculation of differential gene expression.

### Immunoblotting

Cells or EVs aliquots were lysed in RIPA buffer containing protease inhibitors (Sigma, Oakville, Canada). Equal amounts of proteins were resolved on 10% SDS-PAGE and transferred to a nitrocellulose membrane (BioRad). Membranes were blocked in TBS containing 5% non-fat dry milk and exposed overnight at 4 °C to mouse-anti-Vimentin (ab8978, Abcam), rabbit-anti-CDH1 (3195, Cell Signaling), Rabbit anti-GM130 (ab52649, abcam), Mouse anti-TSG101 (ab83, abcam), Mouse anti-Alix (2127, Cell Signaling), or mouse-anti-β-Actin (A5316, Sigma) antibodies. Membranes were washed in TBST (TBS-0.05% Tween-20) and incubated with either anti-rabbit or anti-mouse peroxidase-conjugated secondary antibody (Sigma) for 1 h at room temperature and the blots were developed using Immobilon Western HRP Substrate (Millipore).

### In vivo tumor growth

Five-week-old female NOD-SCID mice (Jackson Laboratory) were used with approval and in compliance with McGill University Health Centre Animal Compliance Office (Protocols 2012–7280 and 2015–7731). Cells growing in log phase were harvested by trypsinization and washed twice with HBSS. Mice (2 to 3 mice) were injected subcutaneously in the right flank with 2 million cells in 200 μl HBSS/Matrigel mixture. The resulting xenotransplants were photographed and processed for immunohistochemistry.

To explore the possibility that EVs could transfer malignant traits to in vivo models, NOD-SCID mice were injected every other day in the tail vein with EVs (~ 4 × 10^6^ particles in 100 μl HBSS) isolated from MC38 cells conditioned medium. Mice received these injections for 5 weeks. Four weeks later, mice were euthanized and the internal organs were harvested for histology and immunohistochemistry analyses.

### Immunohistochemistry labelling procedures

Mice xenotransplants were collected, fixed in 10% buffered formalin and embedded in paraffin. Slices were stained with H&E (hematoxylin and eosin) according to standard protocols and processed for immunohistochemistry. Briefly, 5 μm tissue sections were dewaxed in xylene and rehydrated with distilled water. After antigen unmasking, and blocking of endogenous peroxidase in 3% hydrogen peroxide, the slides were incubated with primary antibodies (Additional file 1: Table S2). Labeling was performed using iView DAB Detection Kit (Ventana) on the Ventana automated immunostainer. Sections were counterstained lightly with Hematoxylin before mounting.

### Karyotype analysis

Standard chromosomes G-banding analysis was performed on mitotic *BRCA1*-KO fibroblasts at the end of the 3 weeks culture period in the presence of cancer EVs. Karyotype was evaluated on a total of 20 metaphases.

### Statistical analysis

Statistical analysis Statistical differences were analyzed using Student’s t test for unpaired samples. An ANOVA followed by the Dunnett test was used for multiple comparisons with one control group. A *p* value < 0.05 was considered statistically significant.

## Results

### Colorectal cancer extracellular vesicles increased *BRCA1*-KO fibroblasts proliferation and reduced their apoptosis

EVs are released into a variety of body fluids in vivo, and into the media of cultured cells in vitro, to accomplish important biological functions [15, 19, 23, 24]. They exert different biological effects by conveying their cargo into target cells [16, 20] and they are reported to intervene in cancer invasion and metastasis via metastatic niche modeling in target organs [20, 25, 26]. Herein, we sought to evaluate the ability of colorectal cancer-derived EVs to transform the *BRCA1*-KO fibroblasts into colon cancer cells. We isolated EVs from a human colorectal adenocarcinoma cell line (i.e. HT-29) and from colorectal cancer patients’ sera (Table 1). First, we confirmed their identity both physically and phenotypically (Additional file 1: Figure S1A-S1C) [27]. As visualized by electron microscopy, and measured by Nanosight tracking analyses, the isolated entities were rounded structures of 50 to 120 nm in diameter. The identity of these EVs was further characterized by labeling for selective markers (i.e. Alix, TSG101) and by confirming the absence of other cell organelle markers (i.e. GM130) to rule out contamination with other cellular components [27].

**Fig. 1:**
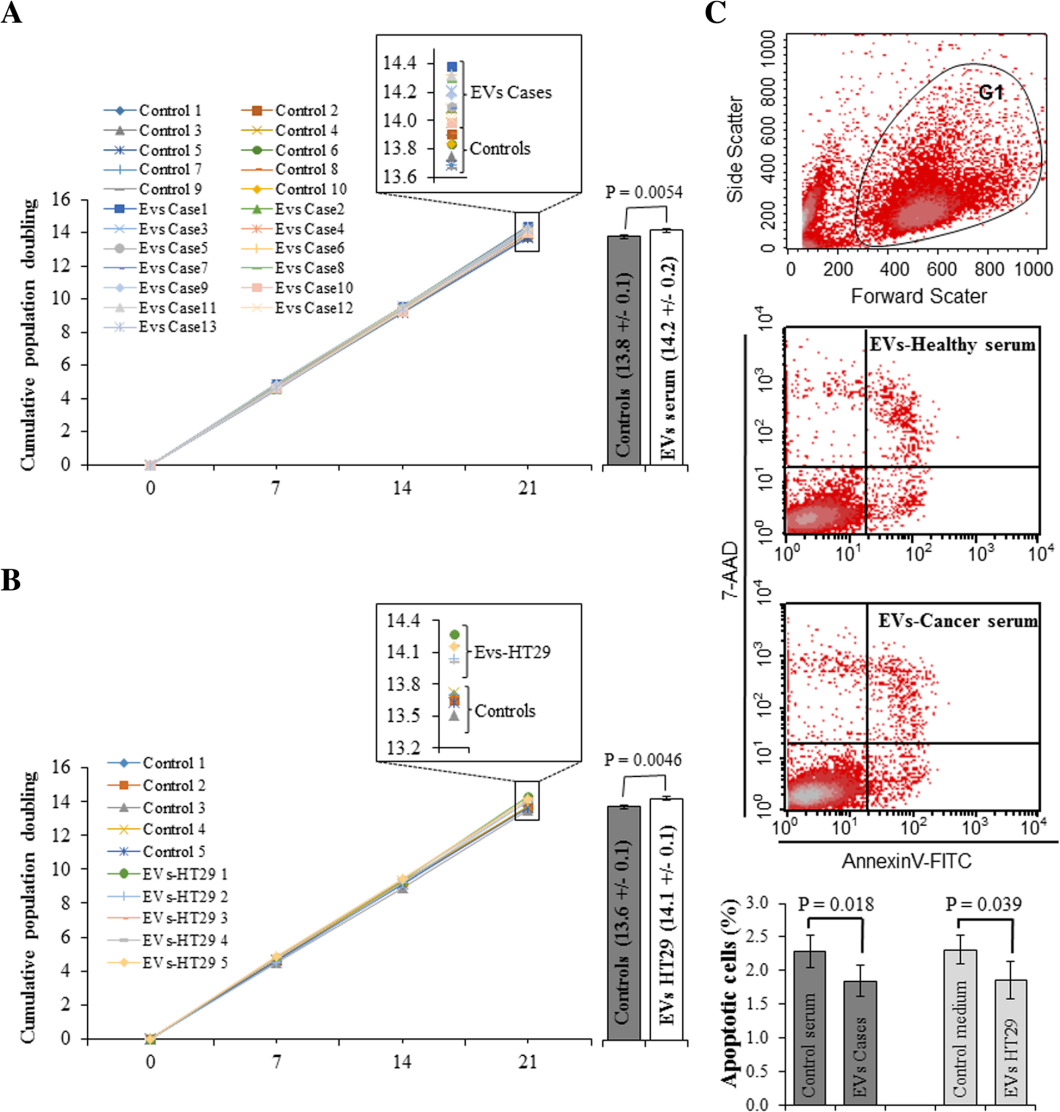
Colorectal cancer EVs increased *BRCA1*-KO fibroblasts proliferation and reduced their cell mortality**.** *BRCA1*-KO fibroblasts were cultured for 3 weeks in the presence of EVs isolated from control human sera, or cancer patient sera (**a**, **c**), and control medium or EVs isolated from HT-29 cell line conditioned medium (**b, c**). Cells were analyzed for their growth potential (**a**, **b**) and cell viability (**c**). **a** and **b** Population doublings capability was calculated at every passage. Data in inserts and column graphs represent cumulative population doublings at the end of the treatment periods. Column graphs represent pooled data from 10 control vs. 13 cancer patient sera (**a**), and from 5 control vs. 5 independent batches of HT29 conditioned media (**b**). Data are mean ± SD. *P* values are represented on the column graphs. (**c**) Treated cells were analyzed for cell viability using AnnexinV and 7-AAD staining followed by flow cytometry analyses. Cells were gated (G1) in a biparametric FSC/SSC graph. The percentage of apoptotic (AnnexinV positive and 7AAD negative) cells were plotted for comparison. Column graphs represent pooled data from 5 control vs. 5 cancer patient sera, and from 4 control vs. 4 independent batches of HT29 conditioned media. Data are mean ± SD. P values are represented on the column graphs

We showed that cancer patient sera increased the proliferation of *BRCA1*-KO fibroblasts [16]. To investigate the behavior of these cells when exposed to cancer-derived EVs, *BRCA1*-KO fibroblasts were treated for 3 weeks and population doubling rate was measured at successive cell passages. Cells treated with cancer serum EVs and cancer cell-derived EVs displayed increased proliferation as judged by the increase in cell population doubling. Increased cumulative population doubling was observed at 2 weeks following the initiation of treatments and became significant by 3 weeks (Fig. 1a and b). In parallel, we verified if cancer EVs affected cell viability in vitro. At the end of the 3-week treatment period, we analyzed cell viability using AnnexinV/7-AAD staining. Treatments with both cancer serum and cancer cell-derived EVs decreased the percentage of apoptotic cells (AnnexinV^+^/7-AAD^−^ cells) (Fig. 1**c**). These data suggest that cancer EVs significantly enhanced cell proliferation and decrease apoptosis in *BRCA1*-KO fibroblasts in vitro.

### Colorectal cancer extracellular vesicles induce a colonic phenotype via transfer of cancer genetic material

To determine the role of cancer EVs in cells transformation, *BRCA1*-KO fibroblasts were treated for 3 weeks with these EVs isolated from cancer serum and conditioned media from cancer cells, and then were transplanted subcutaneously into NOD/SCID mice. To rule-out any possible contamination or carry-over of cancer cells from media, aliquots of the culture medium were placed in a culture plate and incubated at 37 °C, 5% CO2 for 4 weeks. We did not observe any cell growth during this period. In addition, at the end of the treatment period, an aliquot of *BRCA1*-KO fibroblasts exposed to EVs isolated from the serum of a female colorectal cancer patient was karyotyped. G-banding analyses revealed a normal male karyotype of 46,XY (Additional file 1: Figure S1D). This showed that *BRCA1*-KO fibroblasts have a normal karyotype that is not changed following cancer EVs treatments and rules out the possibility that the observed effect was secondary to the presence of cancer cells carried over from conditioned media or patients’ sera.

**Fig. 2:**
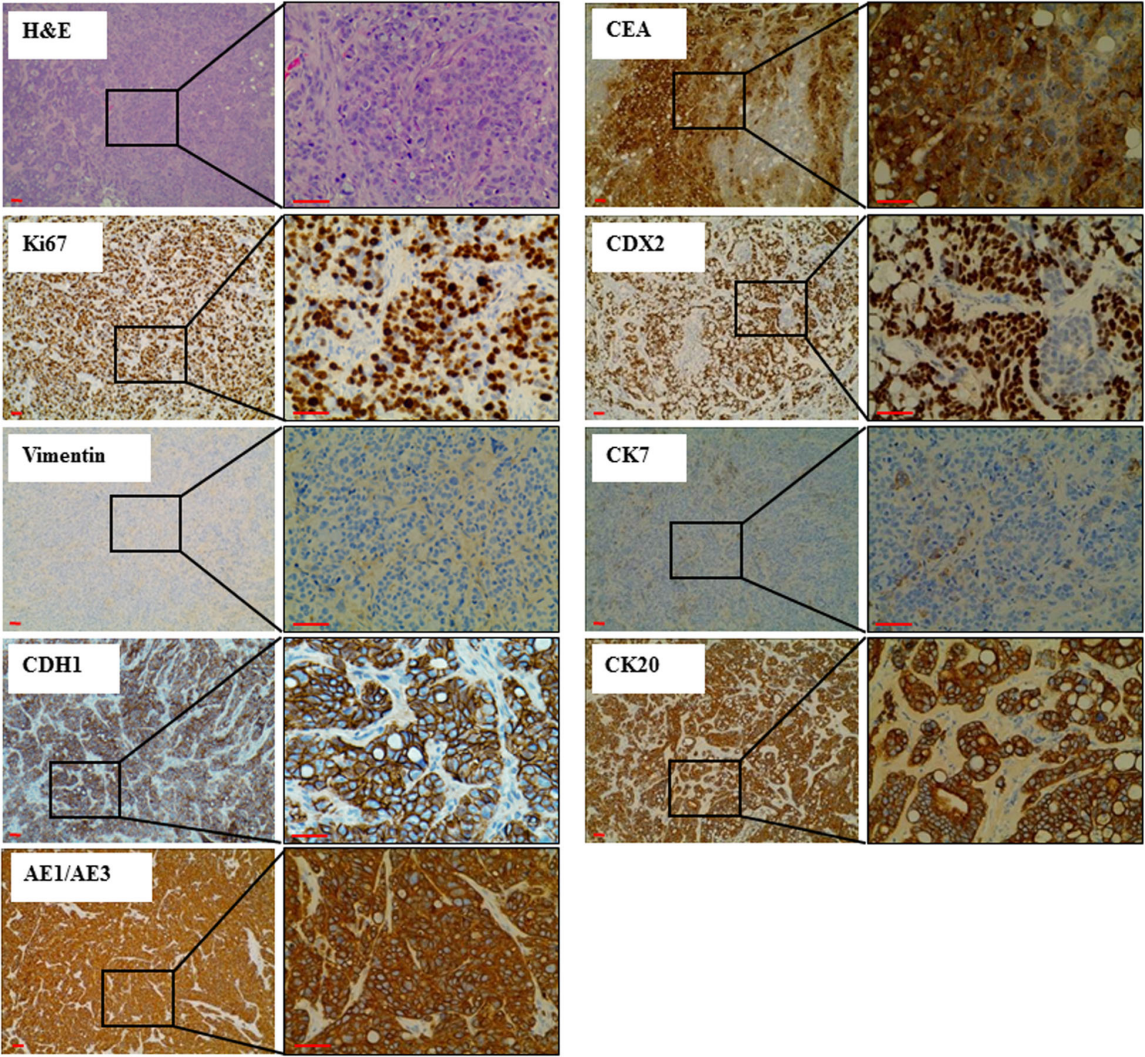
Cancer EVs transfer malignant trait to target cells in vitro. *BRCA1*-KO fibroblasts were treated for 3 weeks with EVs isolated from cancer cells conditioned medium, cancer patient sera or respective controls. Treated cells were injected subcutaneously into NOD/SCID mice that were followed for 4 weeks for tumors growth. Developing tumors were excised, fixed and embedded in paraffin. Tissue sections were processed for H&E staining or immunolabeled with antibodies to Ki67, vimentin, CDH1, CEA, CDX2, CK7, CK20 and AE1/AE3. Representative pictures are shown. Data are representative of five cell cultures exposed to EVs isolated from HT29 cells conditioned medium and thirteen cell cultures exposed to EVs derived from colorectal cancer sera. Scale bars: 50 μm

Mice injected with *BRCA1*-KO fibroblasts treated with control media, or EVs extracted from sera of healthy patients did not develop any subcutaneous masses, whereas all mice injected with *BRCA1*-KO fibroblasts treated with HT29 conditioned medium EVs and EVs extracted from sera of cancer patients developed visible tumors (Table 1). The xenotransplants displayed highly proliferative adenocarcinoma phenotypes as judged by H&E staining and Ki67 labeling (over 85% positive cells) (Fig. 2). These xenotransplants were analyzed by a certified pathologist who confirmed that *BRCA1*-KO fibroblasts displayed colorectal cancer phenotype as judged by the expression of specific markers (CK20, CEA, CDX2, AE1/AE3; Fig. 2). Moreover, the transformed *BRCA1*-KO fibroblasts had changed their fate by adopting an epithelial phenotype as they lost the expression of vimentin and gain the expression of CDH1 (i.e. epithelial cadherin) (Fig. 2). Wild type fibroblasts (i.e. *BRCA1* proficient) did not undergo any malignant transformation after exposure to cancer EVs. These data confirm that cancer EVs transfer their cargo to target *BRCA1*-KO fibroblasts to induce their malignant transformation and suggest that this transformation is achieved through a mesenchymal to epithelial transition.

It should be stressed that the phenotypic shift observed in vivo in exposed *BRCA1*-KO fibroblasts was not acquired during in vitro culture. Immunofluorescence analyses of the 3 weeks exposed cells, showed that while they are Vimentin positive, they were still negative for differentiating markers (i.e. CDX2, CK20, CEA and AE1/AE3) (Additional file 1: Figure S2).

In the course of our study, we compared the migratory potential of *BRCA1*-KO fibroblasts exposed to EVs isolated from the sera of healthy individuals and cancer patients. We used 3 control (Control 11–13) and 5 cancer (Cases 14–18) EVs preparation. Cells were analyzed after 1, 2 or 3 weeks of exposure. Cells treated with cancer EVs did not display any difference when compared to cells exposed to control EVs regarding their migratory potential (Additional file 1: Figure S3). This observation was expected since invasiveness and migratory capabilities are malignant traits that dysplastic cells acquire at the end of their transformation as opposed to increased proliferation and decreased apoptotic abilities, which are the first features that a transforming cell acquires in the metaplastic and dysplastic phases.

In order to deliver their cargo and exert their effects, EVs must be internalized by target cells. We observed that *BRCA1*-KO fibroblasts efficiently uptake EVs that disperse in target cells cytoplasm and form aggregates in the perinuclear regions (Additional file 1: Figure S4A). It is known that uptake of EVs is an active process involving cell surface receptors and EVs surface ligands that facilitate EVs/cell interactions [28, 29]. Mass spectrometry (MS) analyses confirmed (data previously published) that *BRCA1*-KO fibroblasts overexpressed proteins involved in EVs uptake (i.e. dynamin, flotillin, integrins, galectin) (Additional file 1: Figure S4B) and that EVs released from transformed *BRCA1*-KO fibroblasts overexpressed ligands involved in EVs internalization (i.e. laminin, thrombospondin, fibronectin, collagen) (Additional file 1: Figure S4C). Antagonistic blockage of the receptors and their ligands (anti-integrin antibody (ASC-8), Cytostatin [30], heparin [31], RGD (an integrins tripeptide binding site found within fibronectin), and Collagenase I) decreased both the percentage of EVs-internalized cells (i.e. PKH-26 positive cells) to 93% and the amount of internalized EVs (1.5 to 2.6 times) following treatments with the antagonists (Additional file 1: Figure S4D). These data confirm that cancer EVs uptake is an active process involving expression of new receptors and overexpression of some receptors on the membrane of *BRCA1*-KO fibroblasts and their ligands on EVs [15].

**Fig. 3:**
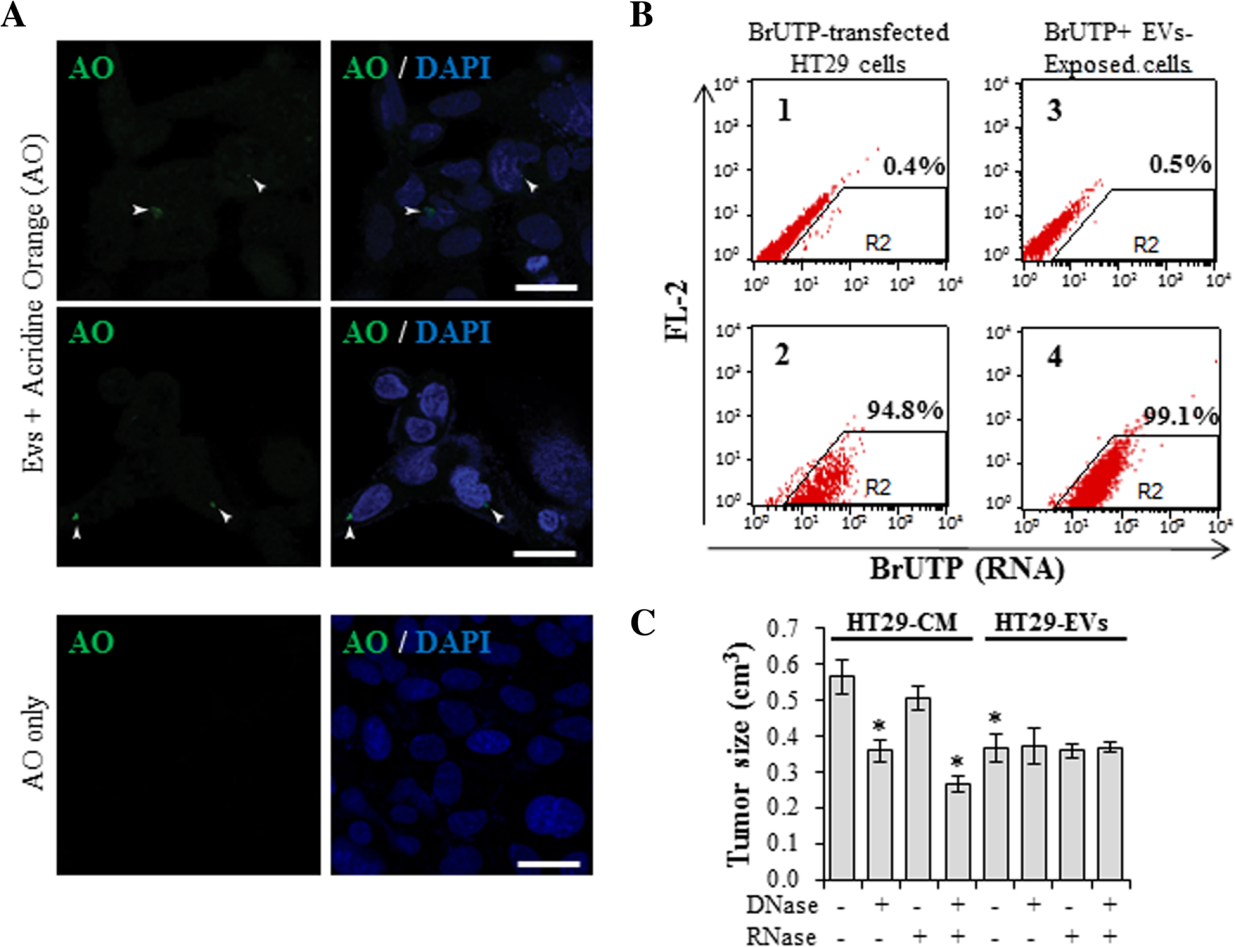
Cancer EVs transferred genetic material cargo to *BRCA1*-KO fibroblasts. **a**, EVs were tagged with acridine orange (AO) to label DNA and *BRCA1*-KO fibroblasts were exposed to these EVs for 24 h. Representative images are shown. Arrowheads point to EV-derived DNA (green) that is entering the nuclei (colored in blue with DAPI). Scale bars: 20 μm. **b**, HT29 cells were transfected with the BrUTP. Aliquotes of transfected (2) or untransfected (1) cells were analyzed for BrUTP incorporation in RNAs. Note that virtually all cells incorporated BrUTP in their RNA (sample 2). Afterwards, conditioned medium was collected from transfected cells for the isolation of EVs. *BRCA1*-KO fibroblasts were exposed (4) or not (3) to these EVs for 12 h. *n* = 2 independent experiments. **c**, HT29 conditioned medium or EVs were treated or left untreated either with DNase, RNase, or both. *BRCA1*-KO fibroblasts were treated for 3 weeks with these media, then treated cells were injected subcutaneously into NOD/SCID mice that were followed for 4 weeks for tumors growth. Data are mean +/− SD. *n* = 2–3 mice. *P < 0.05

After confirming that EVs were responsible for the transformation of the *BRCA1*-KO fibroblasts into colon cancer cells, we sought to investigate the hypothesis that cancer genetic material present in the cancer EVs could be transferred and uptaken by the recipient cells. EVs were tagged with acridine orange (AO when it interacts with dsDNA turns green) [32] to verify whether cancer DNA would be uptaken by the cells. *BRCA1*-KO fibroblasts were exposed to AO-tagged EVs for 24 h and were then analyzed by confocal microscopy. Although AO-tagged DNA positive cells were few (15.6 +/− 3.2%), DNA contained in EVs was still transferred to the target cells nuclei as seen by superimposition with DAPI staining (Fig. 3a). To confirm that even cancer RNA was actively uptaken by *BRCA1*-KO fibroblasts, HT29 cells were transfected with the BrUTP (that substitutes for UTP during RNA transcription) [33]. Conditioned medium was collected from HT29 transfected cells to isolate their EVs that were exposed to *BRCA1*-KO fibroblasts. Both HT29 cells and *BRCA1*-KO fibroblasts were analyzed by flow cytometry to analyze the efficiency of BrUTP transfection, and BrUTP positive RNA transfer, respectively. We observed that almost all transfected HT29 cells incorporated BrUTP in their RNA (Fig. 3b, sample 2). More important we confirmed that all *BRCA1*-KO fibroblasts exposed to transfected HT29 EVs contained BrUTP-labeled RNA (Fig. 3b, sample 4). These data validate our hypothesis that cancer EVs are responsible for the horizontal transfer of neoplastic DNA and RNA to target cells.

To determine the origin (i.e. vesicular vs. extra-vesicular) of the genetic material involved in the transforming abilities of cancer EVs, we treated HT29 cancer cell EVs and the conditioned medium before EVs isolation with DNase, RNase or both. *BRCA1*-KO fibroblasts were exposed to these two different media and analyzed for their tumorigenic potential after subcutaneous injection into NOD-SCID mice (Fig. 3c). While DNase treatment of HT29 conditioned medium reduced the tumorigenic transformation seen in *BRCA1*-KO fibroblasts, RNase treatment had no effect. However, nucleases treatment of cancer EVs had no effect on their ability to transform *BRCA1*-KO fibroblasts, suggesting that the genetic materials involved in the transformation of target cells are protected inside the EV lumen. Altogether, these observations suggest that circulating cancer DNA plays an important role in the transformation of target cells and confirm that this oncogenic genetic material can also be found in the EVs. The EVs might protect this onco information and prevent its destruction when circulating in the blood or other biological fluids.

### Mutated cancer genes are transferred through the extracellular vesicles to the *BRCA1*-KO fibroblasts

EVs carry in their lumen constituents of the original cancer cells that permit intercellular communication via the horizontal transfer of effector bio-molecules (mRNA, non-coding RNA and DNA as mutated and amplified oncogene sequences and retrotransposon elements) [20, 34–37]. Following the observation that cancer EVs transfer RNA and DNA to target *BRCA1*-KO fibroblasts, we sought to further characterize the Evs-contained genetic components and confirm the transfer of colon cancer DNA to the *BRCA1*-KO fibroblasts. We cleared colon cancer EVs from contaminating external DNA and RNA by using DNase and RNaseA digestions, respectively. *BRCA1*-KO fibroblasts were then treated or not with colon cancer EVs for 6 weeks. DNA isolated from all cell cultures and from cancer EVs were then processed for high throughout whole-genome sequencing (Additional file 1: Figure S5).

After completion of the bioinformatics analysis, whole-genome sequencing showed that EVs DNA covered 95–97% of the human genome at a mean depth of 6–9× at high-quality sequencing with 80–86% of bases having greater than or equal to sequencing quality scores of Q20 (Additional file 1: Figure S5). These data confirm that EVs DNA was representative of the entire human genome.

We identified a list of 68,649 candidate allelic variants, which were found in the EVs DNA sample and in the *BRCA1*-KO fibroblasts exposed to the cancer EVs, but not found in the control *BRCA1*-KO fibroblast sample. From the above list, we extracted 268 synonymous SNV and nonsynonymous SNV variants. After visual inspection of the alignment (BAM) files of the *BRCA1*-KO fibroblast sample, we excluded those variants whose reads show more than two non-reference alleles and not confirmed by at least 5 reads. With this quality check, we confirmed that 58 of 268 exonic variants were absent in the control *BRCA1*-KO fibroblast sample (Additional file 1: Table S3). Also, we scanned these variants for amino-acids sequence changes, and we found that 147 of them displayed mutations that translated into a change in the amino-acids sequence. This data endorse the hypothesis that mutated cancer genes are transferred to the *BRCA1*-KO fibroblasts and suggest that they might play a role in their malignant transformation.

### Transferred mutated cancer genes are transcribed in the *BRCA1*-KO fibroblasts

To verify the hypothesis that the mutated cancer genes that were transferred through the EVs might be transcribed by recipient *BRCA1*-KO fibroblasts, we performed RNA sequencing on colon cancer EVs, and *BRCA1*-KO fibroblasts prior and after exposure to colon cancer EVs.

In order to obtain a high-quality set of RNA variants, the following filters with the Vcftools software were applied to the call set of one colon cancer EVs sample, three merged *BRCA1*-KO fibroblasts samples and three merged exposed *BRCA1*-KO fibroblast samples: minDP 10, minGQ 10, minQ 30. Indels were not considered for the following analysis.

Overall, we identified 75,163 SNV in the colon cancer EVs sample, 152,567 SNV in the merged *BRCA1*-KO fibroblasts samples and 154,729 SNV in the merged exposed *BRCA1*-KO fibroblasts samples. Control and exposed *BRCA1*-KO fibroblasts showed 101,508 SNV in common. Notably, 9063 SNV were present in the colon cancer EVs RNA and in the exposed *BRCA1*-KO fibroblast RNA but not in control *BRCA1*-KO fibroblasts. Out of these variants, 918 were exonic. We scanned these variants for amino-acids sequence changes, and we found that 380 of them displayed mutations that translated into a change in the amino-acids sequence.

Five mutated gene variants that were transferred to the *BRCA1*-KO fibroblasts (BTN3A2, DDX51, LARP6, AP1G1 and COL18A1) were found to be also present as RNA variants following RNA sequencing of *BRCA1*-KO fibroblasts after exposure to cancer EVs. Three of them (BTN3A2, AP1G1 and COL18A1) displayed mutations that translated into a change in the amino-acids sequence. These variants were not present in the *BRCA1*-KO fibroblasts prior to exposure.

Furthermore, by comparing the variant set of the three samples identified from RNA sequencing variant calling, we identified 30 loci that were polymorphic in EVs and exposed *BRCA1*-KO fibroblasts but not in control *BRCA1*-KO fibroblasts (Table 2). Moreover, in about 80 different positions the alternative allele was confirmed by one single base only (while present in the majority of reads in EVs and exposed *BRCA1*-KO fibroblast RNA). All these findings seem to suggest that EVs DNA might be actively transcribed in exposed *BRCA1*-KO fibroblasts.

**Table 2:** Comparison of identified RNA variants based on loci polymorphism

						Reads	
Chr	Position	Ref.	Function	Gene ID	EVs	Fibro	Fibro + EVs
chr1	91985092	A	intronic	CDC7	..tt.,	.,,..,	...T..T
chr1	247370225	C	intergenic	MIR3916;VN1R5	tT.TTTT	,,,,.	T.TTtT
chr1	153907839	T	intronic	DENND4B	.CC.$A..C.C.	.....	CC.,Cc
chr2	232999492	A	intronic	DIS3L2	...,GG,,.......	.,,..,..	.,,.Gg..
chr2	37343160	C	intronic	EIF2AK2	...tt	,.,...	..,$.,TT....tT.
chr2	233429254	A	intronic	EIF4E2	.....GGG..G	,.,..	..GG,
chr3	196820370	T	intronic	DLG1	G.GGG...GG	.....,.	G.G.G
chr3	156466817	G	exonic	LINC00886	...C....C.C,.	.,,,....	C.Cc.C.
chr4	128934795	G	intronic	ABHD18	..AAa	...,...,	a.aAA,
chr4	48172266	G	exonic	TEC	Aa...	.....,,	.........,,,
chr5	147516598	T	UTR3	SPINK5	.c,CCCCC	.....,,,	...CCC.,,,C..,,,,.,
chr5	133311246	G	intronic	VDAC1	.A.AA.	.$...	.,$.aAA.A,.A
chr6	28060485	G	exonic	ZSCAN12P1	CCcC.	...,,..,,.	.C.C,.CC.c,C.,CC.,C
chr7	2647007	G	UTR3	IQCE	a.A,Aa,	...,.,..,..	,.,.AaAAAA.A.A,a
chr7	76252581	G	intronic	LOC100133091	.TTTTTt.T	......	TTTTTt..,..TtT
chr7	66042937	G	intronic	GS1-124 K5.11	c,,,c	,,.....	C.c.c.c,c
chr8	131069787	C	intronic	ASAP1	T..TT....	....,.,....	.,..T,T
chr10	27474817	G	intronic	MASTL	A,$.,,,a,a,a,	.,..,...	A.,,.aa,,.,.
chr10	134540571	C	intronic	INPP5A	T.t.T	..,,,.,,	...,tT
chr10	33609449	T	intronic	NRP1	aaaa,a	.....	AAAAaA,
chr11	33061738	C	UTR5	TCP11L1	TTTTTttTTtTTt	...,.....,,	TTtTTTTT
chr12	13134982	C	intronic	LOC100506314	.G.GG,G,^S.	..,,.,...	GG....,.Gg.,.,
chr12	71518329	G	downstream	TSPAN8	.TTTT	.,.,,	TT,,,.....,^S.
chr12	56373847	C	intronic	RAB5B	.TTTTT,,.,TTTT	.,...	TTT.....
chr12	106741689	C	downstream	TCP11L2	..,,,,..	...,,.,...,	.T,,,,,,,....,,^S.^S.
chr14	105608086	A	UTR3	JAG2	.,..,.	...,,.....	,.....,..........T.
chr15	69703990	A	intronic	LOC145694	.gGg.,G	.,....	G.,..G,.
chr16	56874857	C	intronic	NUP93	T.TTT	......	T..Tt.TTT
chr17	38341874	A	intronic	RAPGEFL1	..G,G,	,...,,	..,,..,GG...g.,.
chr19	49832810	T	intergenic	SLC6A16;CD37	C..,.CC	..,,,	.......Cc.,..C,cC
chr19	49835569	T	intergenic	SLC6A16;CD37	C.c..	....,....,,	..C.C...C.C.CC.C.,..,c,
chr19	45993568	A	intronic	RTN2	CcCcc	...,..	CCC.C..,C...,.CC

*Chr* chromosomes, *Position* chromosomic position, *Ref.* reference nucleotide at the chromosomic position

### Messenger RNA (mRNA) profiling reveals that cancer extracellular vesicles actively transfer transcripts involved in the regulation of cell growth and survival, and induction of MET phenotype

**Fig. 4:**
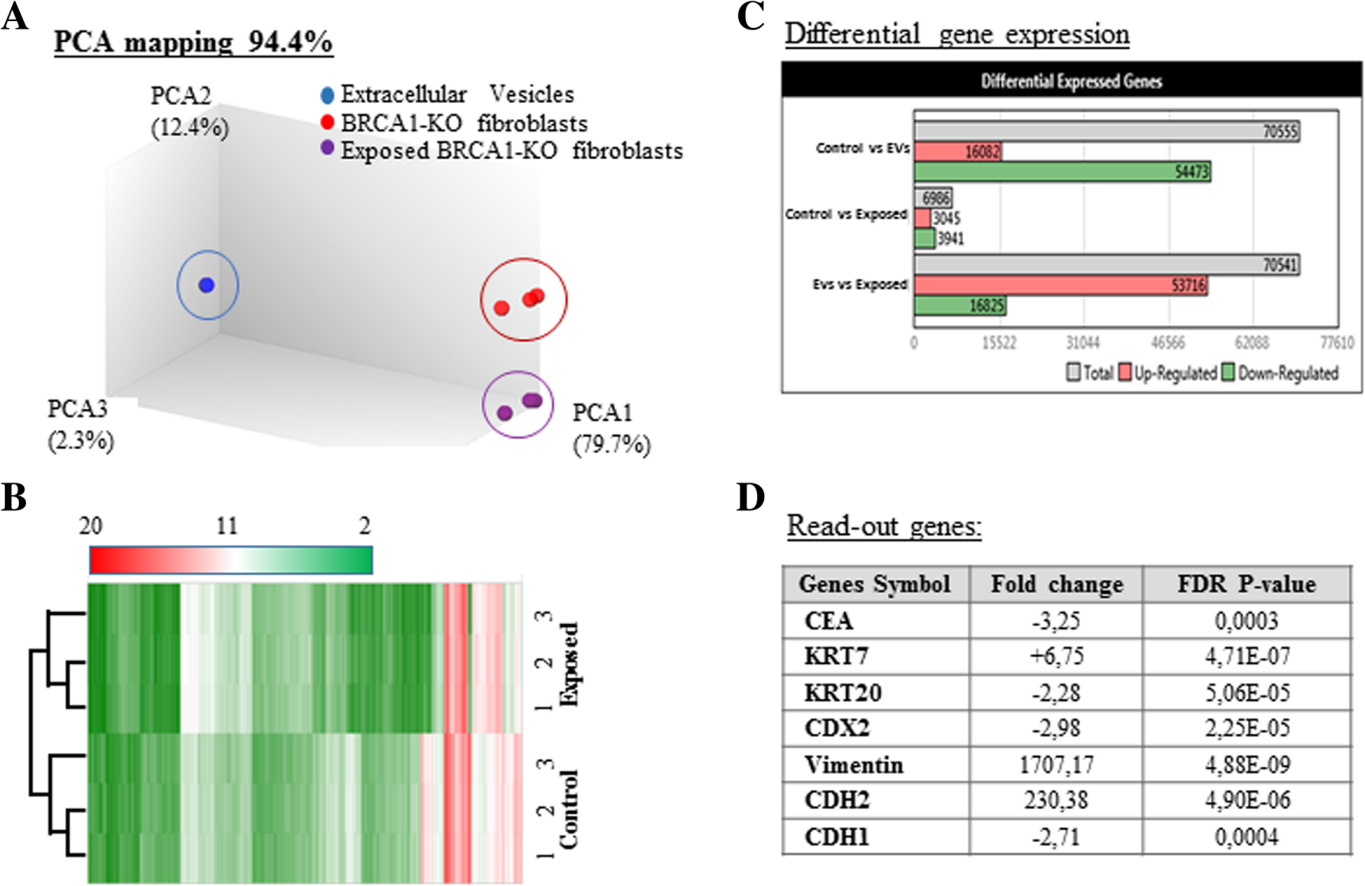
Exposure of *BRCA1*-KO fibroblasts to cancer EVs changed their transcriptome profile. **a** and **b**, PCA mapping and hierarchical clustering showed that *BRCA1*-KO fibroblasts exposed to EVs clustered differently from non-exposed cells. In addition, EVs clustered far from cells. **c**, Differential gene expression between EVs-exposed and non-exposed cells. Data were obtained with 3 independent cell cultures. Filter criteria were set as follows: Fold change: > 2 or < − 2 and FDR *P* value < 0.05. **d**, Accuracy of the microarray data mining. List of read-out genes based on immunohistochemical labeling data (Fig. 1)

In parallel to RNA-Seq mining, we performed mRNA array analyses to search for transcripts differentially expressed in *BRCA1*-KO fibroblasts prior and following exposure to cancer EVs. We used the Transcriptome Analysis Console to analyze the differential gene expression. Data quality control showed that templates labeling and hybridization were normal and uniform. Also, the PCA mapping and hierarchical clustering showed that samples clustered differently (Fig. 4a and b). We identified 6986 transcripts differently expressed between cancer EVs-exposed and non-exposed *BRCA1*-KO fibroblasts. Out of these transcripts, 3045 were downregulated and 3941 transcripts were upregulated in cancer EVs-exposed *BRCA1*-KO fibroblasts (Fig. 4c and Additional file 1: Figure S6A).

**Fig. 5:**
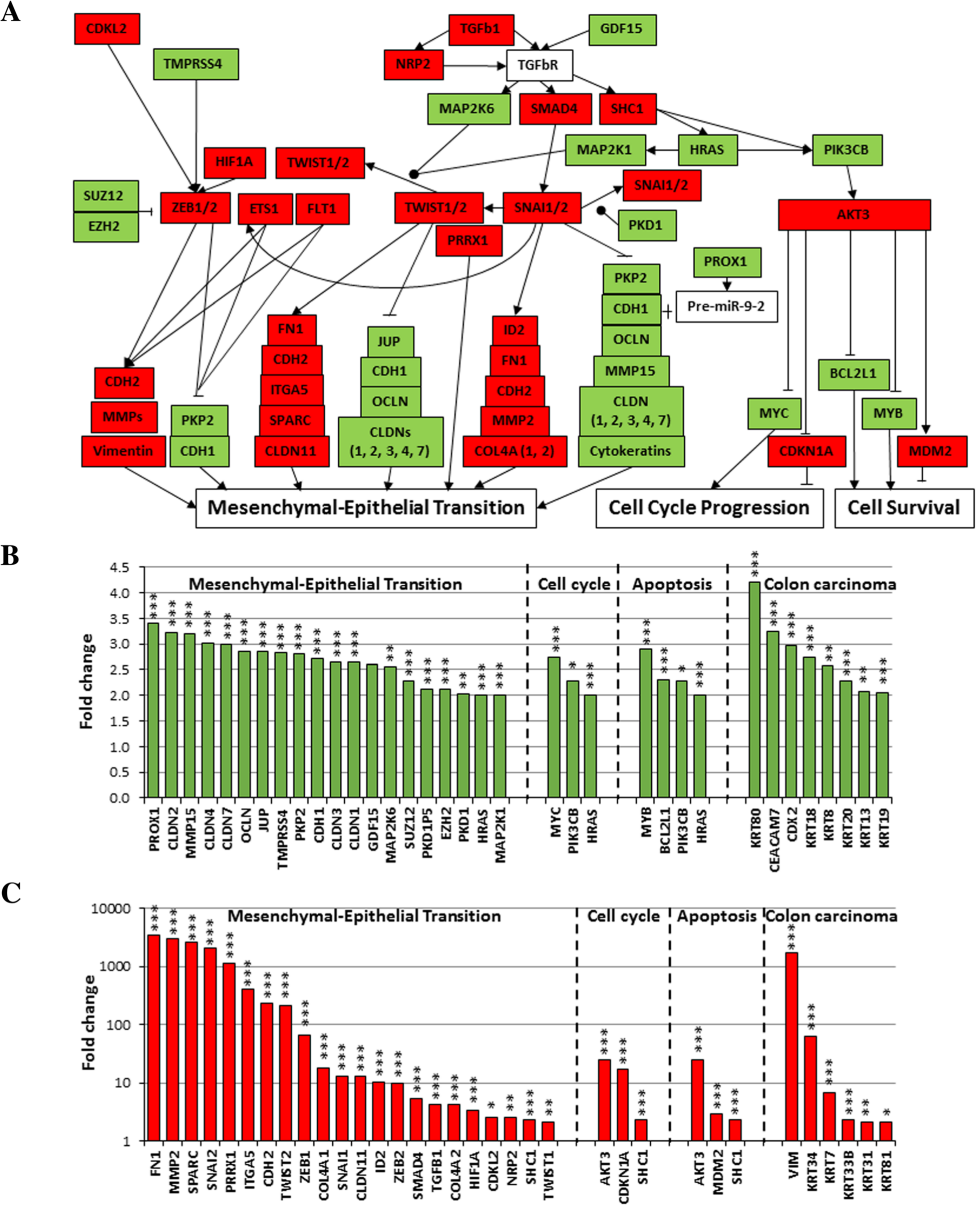
*BRCA1*-KO fibroblasts exposed to cancer cells EVs undergo Mesenchymal to Epithelial Transition (MET), increased proliferation and reduced apoptosis. *BRCA1*-KO fibroblasts were treated for 3 weeks with EVs isolated from HT29 cancer cells conditioned medium. RNA was isolated and processed for microarray analyses. **a**, Three major pathways involving genes implicated in MET, cell cycle progression and cell survival are shown. Green and Red highlight up-regulated and down-regulated genes in EVs-exposed cells, respectively. **b** and **c**, Up-regulated (**b**) and down-regulated (**c**) genes in EVs-exposed cells. Genes expression with a cut-off of − 2 and 2 and a FDR P value < 0.05 are shown. *n* = 3 independent cell cultures. ● phosphorylation. * *P* < 0.05, ** *P* < 0.01, *** *P* < 0.001

Since we had observed that xenotransplants obtained with cancer EVs-treated *BRCA1*-KO fibroblasts displayed colorectal cancer epithelial phenotype, as they were positive for CEA, CDX2, CK20 and CDH1, and negative for CK7 and vimentin (Fig. 2), we used this documented event as a readout for the accuracy of our microarray data mining. We found that this pattern of phenotypical expression was also mirrored at the mRNA levels (Fig. 4d). WikiPathway analyses showed that the differentially expressed genes were involved in cellular functions, mainly cellular growth and senescence, apoptosis signaling, endoderm and colon carcinoma differentiation, and MET transition (Fig. 5a-c and Additional file 1: Table S4).

**Fig. 6:**
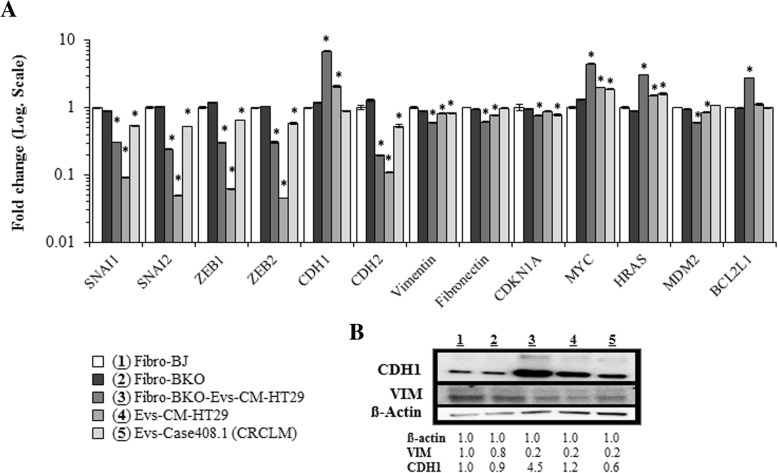
Validation of differential expression of genes involved in the MET process. **a**, Naïve fibroblasts (BJ) (1), *BRCA1*-KO fibroblasts untreated (2) or treated with EVs isolated from HT29 cancer cells conditioned medium (3), and EVs isolated from HT29 cells conditioned media (4) or colorectal cancer patient serum (4) were analyzed for the expression of mesenchymal (SNAI, ZEB, CDH2, Vimentin and Fibronectin), epithelial (CDH1), cell cycle regulators (CDKN1A, MYC and HRAS), and apoptosis regulators (MDM2 and BCL2L1) markers by qPCR. Results are representative of one experiment performed twice. Data are mean +/− SD. *n* = 2. *P < 0.05. Note that data are expressed in Log. Scale. **b**, The same samples as in (**a**) were analyzed by immunoblot for the expression of mesenchymal (Vimentin; VIM) and epithelial (CDH1) markers. β-actin is used as calibrator for proteins loading. The table shows proteins levels in the corresponding samples. *n* = 2 independent experiments

We validated the differential gene expression at both RNA and protein levels by choosing genes involved in the MET process (Fig. 6a and b), cell growth and cell death (Fig. 6a). qPCR analyses revealed that the mesenchymal markers (i.e. SNAI1, SNAI2, ZEB1, ZEB2, CDH2, vimentin and fibronectin) expression was decreased in cancer EVs-exposed *BRCA1*-KO fibroblasts, while the expression of CDH1 was increased (Fig. 6a). In parallel with the increased expression of the CDH1 protein, which is the hallmark of an epithelial phenotype, the expression of the mesenchymal marker vimentin was decreased (Fig. 6b and Additional file 1: Figure S7). In addition, while the expression of the cell cycle progression inhibitor CDKN1A (p21) was decreased in cancer EVs-exposed cells, the expression of the oncogenes MYC and HRAS was increased (Fig. 6a). Finally, while the expression of the cell death inducer MDM2 was decreased in cancer EVs-exposed cells, the expression of the anti-apoptotic factor BCL2L1 was increased (Fig. 6a). This observation strongly suggests that colon cancer mRNA transferred through the EVs is able to modulate the expression of different sets of transcription factors that induce a change in the fate of the fibroblast through the activation of the MET process, and regulation of cell growth and apoptosis.

To rule out the possibility that the acquisition of the epithelial phenotype in the fibroblasts was an effect due to *BRCA1* knock-out, rather than an effect secondary to horizontal transfer of cancer mRNA, we analyzed the expression of SNAI1, SNAI2, ZEB1, ZEB2, CDH2, vimentin, fibronectin, and CDH1 in naïve fibroblasts. The results were then compared to the results obtained in *BRCA1*-KO fibroblasts (Fig. 6a and b). No difference was found in the expression of both the mesenchymal and epithelial markers between the naïve fibroblasts and the *BRCA1*-KO counterparts proving that this transition occurred later, after cancer EVs exposure. These data suggest that a modulation of the mRNA expression towards a MET phenotype underlie the malignant transformation of target *BRCA1*-KO fibroblasts following exposure to colon cancer EVs.

### Cancer extracellular vesicles-treated *BRCA1*-KO fibroblasts displayed deregulated miRNA expression profile typical of colorectal cancer progression and invasion

**Fig. 7:**
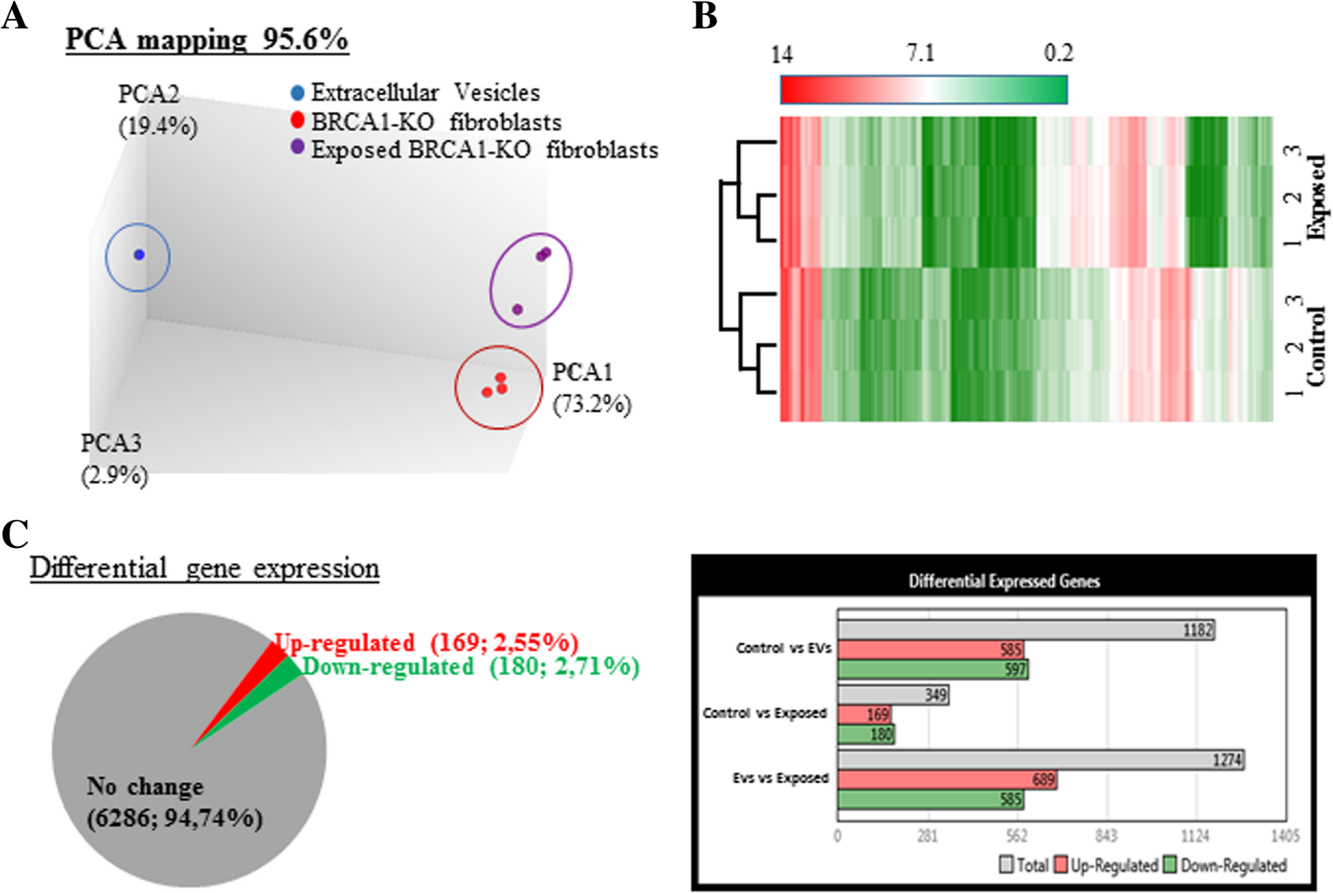
Exposure of *BRCA1*-KO fibroblasts to cancer EVs changed their pattern of miRNA expression. **a** and **b**, PCA mapping and hierarchical clustering showed that *BRCA1*-KO fibroblasts exposed to cancer EVs clustered differently from non-exposed cells. In addition, EVs clustered far from cells. **c**, Differential miRNA expression between EVs-exposed and non-exposed cells. Data are obtained with 3 independent cell cultures. Filter criteria were set as follows: Fold change: > 2 or < − 2 and FDR P value < 0.05

MicroRNAs repress target genes translation and induce rapid degradation of mRNA. EVs contained miRNA that have been reported to regulate tumorigenesis [20, 23, 24, 26]. In order to better understand the mechanisms underlying the malignant transformation of *BRCA1*-KO fibroblasts into colon cancer cells, we further deepened the RNA analyses by profiling differential miRNA expression between *BRCA1*-KO fibroblasts prior and after exposure to colon cancer EVs. The PCA mapping and hierarchical clustering showed that samples clustered differently (Fig. 7a and b). The expression of 349 miRNAs was aberrant in *BRCA1*-KO fibroblasts following treatment with cancer EVs, of which 169 miRNAs were down expressed and 180 miRNAs were over-expressed in cancer EVs-exposed *BRCA1*-KO fibroblasts (Fig. 7c and Additional file 1: Figure S6B). A cross comparison analysis between cancer EVs-exposed and non-exposed cells, revealed a correlation between 152 of the over expressed miRNAs and a down regulation of their mRNA targets. Strikingly, we found that several aberrantly expressed miRNAs are known to be involved in colorectal cancer progression and invasion (i.e. miRNA-193a, miRNA-345, Let7, miRNA-1229, miRNA-1246, miRNA-150, miRNA-21, miRNA-17, miRNA-19) [38–41].

**Fig. 8:**
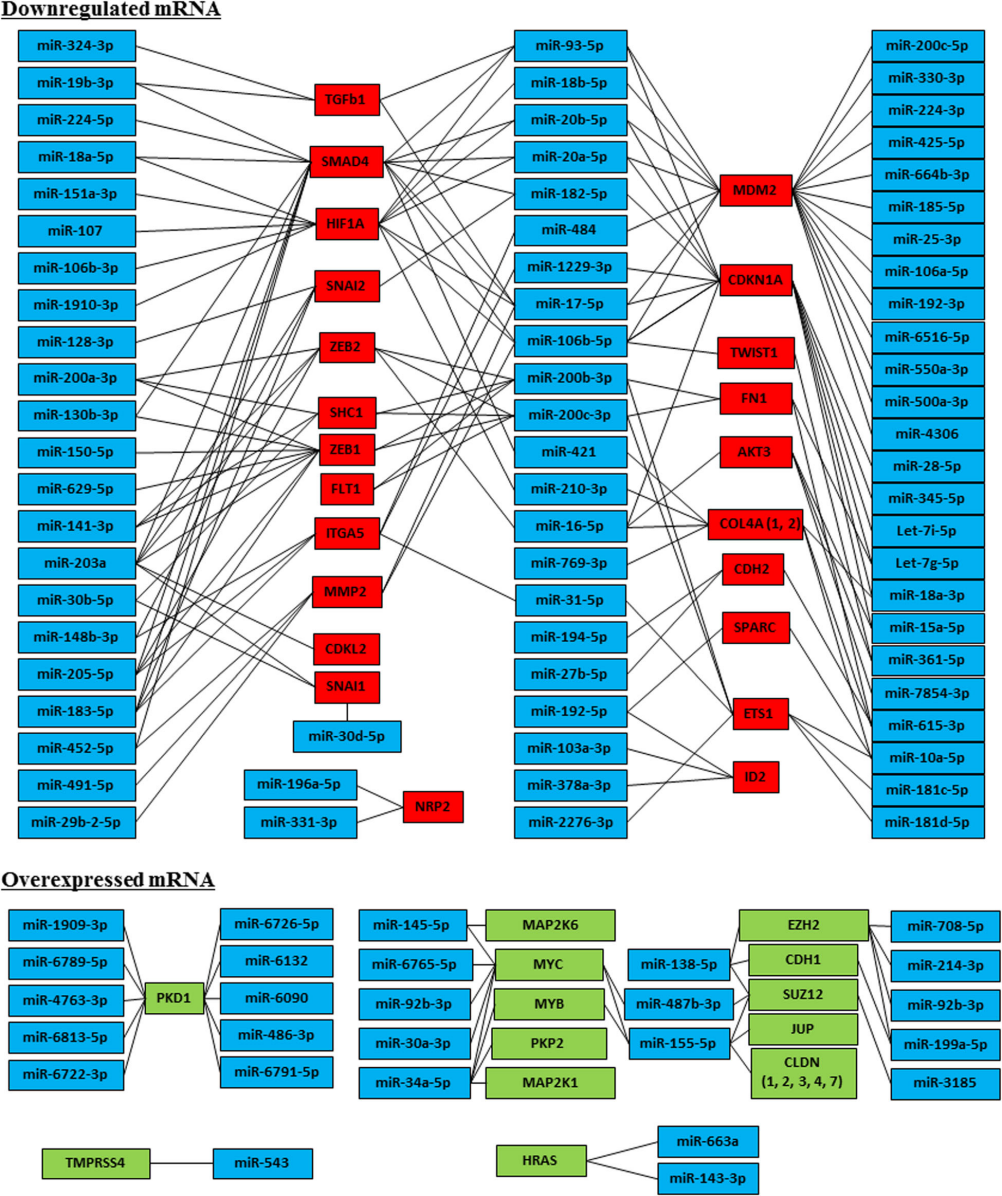
mRNA/miRNA interaction network derived from the microarray analyses describing the transcripts involved in MET process. Connections were retrieved from mirtarbase database (http://mirtarbase.mbc.nctu.edu.tw). Genes expression with a cut-off of 2 are shown. *n* = 3 independent cell cultures. See online Additional file 1: Tables S4 and S5 for the levels of expression

Interaction networks between miRNA and mRNA impacts several biological processes by modulating the expression of proteins. We scanned our data in the mirtarbase database (http://mirtarbase.mbc.nctu.edu.tw) for miRNA target gene predictions and we found that the differentially expressed miRNAs were already known to be involved in many cellular functions. Following this observation, we performed an interaction network analysis to connect the dysregulated miRNA expression in cancer EVs-treated *BRCA1*-KO fibroblasts to the regulated transcripts. We focused our analyses on those involved in MET process (Fig. 8 and Additional file 1: Table S5) and found that overexpression of the epithelial cadherin CDH1 transcripts was concomitant to decreased expression of SNAI and ZEB family members (Figs. 5b, c and 8). As already mentioned, the SNAI and ZEB family members are known regulators of mesenchymal state through regulation of CDH1. The downregulation of their expression causes an upregulation of CDH1 with acquisition of an epithelial phenotype [7]. This modulation in the expression of SNAI and ZEB family members is tightly regulated by several miRNA [42]. Strikingly enough we found that the expression of miRNA30, miRNA203, miRNA128, miRNA148, miRNA183 and miRNA182 (targeting SNAI1 and SNAI2), miRNA16, miRNA200, miRNA141, miRNA203, miRNA205 and miRNA183 (targeting ZEB1 and ZEB2), miRNA17, miRNA16, miRNA378, miRNA1287 and miRNA 615 (targeting vimentin) and miRNA615, miRNA27 and miRNA194 (targeting CDH2) was upregulated while this of miRNA138 and miRNA199 (targeting CDH1) was downregulated in *BRCA1*-KO fibroblasts after exposure to cancer EVs (Fig. 8 and Additional file 1: Table S5). This discovery suggests that colon cancer miRNAs transferred through EVs to the *BRCA1*-KO fibroblasts might be involved in the induction of an epithelial phenotype and acquisition of metastatic features in *BRCA1*-KO fibroblasts.

### Cancer EVs transfer malignant traits in vivo

**Fig. 9:**
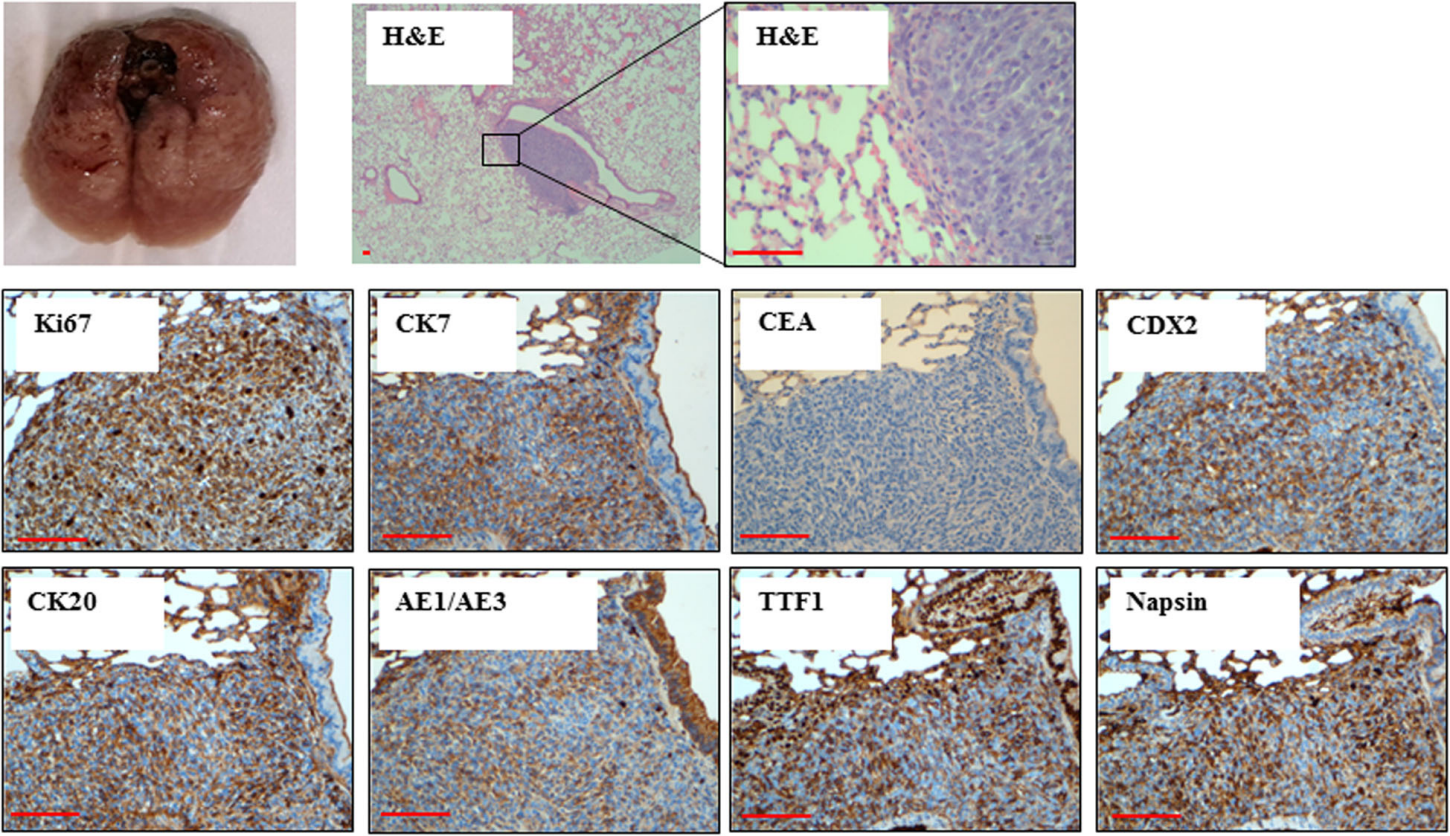
Cancer EVs transfer malignant traits in vivo. NOD-SCID mice were injected every other day in the tail vein with EVs isolated from MC38 cells conditioned medium for 5 weeks. Four weeks later, lungs were harvested photographed and analyzed. Tissue sections were processed for H&E staining or immunolabeled with antibodies to Ki67, CEA, CDX2, CK7, CK20, AE1/AE3, TTF1 and Napsin. Representative pictures are shown. Data are representative of staining perform on lungs harvested from three exposed mice. Scale bars: 50 μm

To determine whether cancer EVs have the ability to transform target cells in vivo, we injected EVs isolated from the murine colon adenocarcinoma MC38 cells via the tail vein to NOD-SCID mice. Two months later, mice developed respiratory distress. At euthanasia, we observed that these mice displayed neoplastic lesions in the lungs (Fig. 9). Developing tumors had proliferative adenocarcinoma phenotypes as judged by H&E staining and Ki67 labeling (10% positive cells) (Fig. 9). Moreover, immunohistochemical staining showed that these lesions were positive for poorly differentiated colon cancer markers (CK20, CDX2, AE1/AE3), and lung cancer markers (TTF1 and Napsin) (Fig. 9). As we already hypothesized and proved in vitro [43], this finding suggests that cancer EVs may have targeted two different cell populations and induced their independent transformation. These data bring more evidence that circulating EVs shed by cancer cells possess the ability to transform target cells at distance even in vivo and strengthens our hypothesis that the malignant transformation of uptaking cells might not be due solely to the messages carried by the cancer EVs but it is also determined by the type of cells that uptake, transcribe and translate the onco-message (mesenchymal vs epithelial cells).

## Discussion

The amount of circulating EVs released by the primary tumor increases with advancing stage of cancer [19]. This evidence suggests a potential role of EVs in cancer progression and invasion. The functional role of EV cargo in cancer biology is currently intensively investigated, as cancer EVs carry genetic material and proteins that favor tumor growth and alter the tumor niche micro-environment [25, 40, 41, 44–48]. Here we provide evidence that colon cancer EVs actively transfer both mutated cancer genes to target cells as well as a bulk of coding and non-coding RNAs acting as modulators of essential cellular pathways that impact cancer growth and progression.

We showed that only around 16% of exposed BRCA1-KO fibroblasts contained colon cancer DNA as opposed to cancer RNA that was taken up by almost all exposed BRCA1-KO fibroblasts (Fig. 3a vs. Figure 3b). This finding favors the hypothesis that a subset of EVs contains DNA and weakens the hypothesis of a selective uptake of cancer EVs as a way to explain the lower number of cells containing foreign DNA [35].

We observed that out of 58 DNA variants transferred to the BRCA1-KO fibroblasts, several included genes that codify for HLA proteins (HLA-DRB5 and HLA-DQA2) and specifically for the extra-membrane portion of some of them. In this in vitro model, the transfer of mutated cancer genes that codify for regions of the HLA system closely associated to areas involved in immunological recognition pave ways to the hypothesis that horizontal transfer of malignant traits might also include the transfer of the ability possessed by cancer cells to escape immune system control, through alteration of the membrane HLA proteins. Further studies addressing the validity of this observation are needed to confirm this assumption that at the moment must be considered and regarded as a pure theoretical possibility.

The microarray analyses performed in this study confirmed the presence of several messenger RNA and non-coding RNAs in the colon cancer EVs and established that the expression of these transcripts was altered in target *BRCA1*-KO fibroblasts following exposure to the same EVs. The observed changes in mRNA levels, in our opinion, might be either secondary to direct transfer of EV cargo (as shown in Fig. 3b), or may be due to transcriptional regulation of these transcripts in target cells following uptake of the cancer EV cargo. We believe that the deregulated expression of these transcripts seen in *BRCA1*-KO fibroblasts could be due to a post-transcriptional event involving miRNA-mediated repression of target genes translation and to induction of degradation of mRNA [21]. Further corroborating this notion, 152 over-expressed miRNAs were present in colon cancer EVs and the uptake of these miRNAs was correlated with down-regulation of their respective mRNA targets in the transformed *BRCA1*-KO fibroblasts. Notably several of these miRNAs had been reported to be involved in colorectal cancer progression and invasion [38–41], strengthening our belief that our in vitro findings might have physiological relevance and probably be one of the mechanisms involved in the metastatic disorder. We showed immunohistochemical evidence that the fibroblasts turn into colon cancer cells and undergo MET while becoming malignant. The evidence that colon cancer EVs contain miRNAs directly involved in the regulation of the transcription factors involved in the MET process and the transcriptomic confirmation of a distinct modulation of the expression of the genomic pattern of the mesenchymal nature of the fibroblasts into an epithelial genotype suggest that the horizontal transfer is, at least in vitro, a formidable mechanism that needs to be understood in depth for its potential in vivo implications.

The hypothesis that metastatic disease might be due not only to circulating cells but also to circulating cancer factors able to transform target cells into cancer cells was brought forward by Garcia Olmo in the late nineties [12] and later on in 2014 by our group [17]. Subsequently, in different studies we established the legitimacy of the hypothesis by showing that *BRCA1*-KO fibroblasts would turn into colon cancer cells when exposed to EVs isolated either from sera of patients with colon cancer or EVs isolated from conditioned media of a colon cancer cell line [15]. Although these results were intriguing, the lack of mechanistic data supporting the theory of a horizontal transfer of malignant traits prevented this theory from drawing the necessary attention from the scientific community.

The results presented in this study for the first time shed new insights on the possible mechanisms involved in the in vitro transformation of the *BRCA1*-KO fibroblasts into colon cancer cells and establish that the concept of horizontal transfer of malignant traits is a phenomenon that deserves to be studied and understood in its entirety. We confirmed that cancer EVs contain genomic DNA spanning all human chromosomes [34, 35, 37] and we observed that several mutated cancer genes were transferred to target *BRCA1*-KO fibroblasts. The presence of mutated colon cancer genes in onco-suppressor-mutated fibroblasts that are in the process of completing a full phenotypical transformation into colon cancer cells is an association that warrants further investigations before discarding this phenomenon as a meaningless association.

The complete reprogramming of benign mesenchymal cells such as fibroblasts into malignant colonic epithelial cells caused by the uptake of only colon cancer EVs is an extraordinary feat. Furthermore, if it is framed within the well-known concept that the activation of only four genes can prompt a full dedifferentiation of human fibroblasts into induced pluripotent stem cells [49], then the possibility that this phenomenon might have an important role in metastatic disease might not be far-fetched.

The consistent differences in genomic pattern between primary and metastatic cancer cells as well as a lack of a clear understanding on how cancer cells manage to apply substantial and reversible phenotypical changes that allow them to escape the tissues of origin, migrate into the lymphatic and blood circulation and home to different organs pose logical doubts on the true identity of the metastatic cells. Our data demonstrates clearly that primed cells can uptake genetic material contained in cancer EVs and indicates that this horizontal transfer of genetic material might be associated with transcription of cancer DNA in the target cell. Although our study and works from other groups showed that cancer cell-derived EVs and cancer patient-derived circulating EVs orchestrate the process of metastasis [25, 26, 50], well-designed in vivo studies are required to unequivocally address the systemic role of EVs in cancer burden and pathophysiology [25, 51].

Cancer EV migration rather than migration of cancer cells might overcome some limitations displayed by the conventional seed to soil model when trying to understand metastatic disease. The in vitro evidence shown here indicates that cancer genetic material can enter primed cells and hijack the replicative machinery of the same cells to induce stunning biological changes. Although we did not give definite confirmation that this cancer genetic material is responsible for the transformation of fibroblasts into colon cancer cells, it must be conceded that, according to conventional knowledge, phenotypes are determined by genotypes and therefore the dramatic modifications that lead a benign mesenchymal cell to turn into a malignant epithelial cell must be necessarily driven by critical genomic changes that are undoubtedly observed in the *BRCA1*-KO fibroblasts after exposure to colon cancer EVs. Extensive sequencing and lineage tracing studies are warranted to definitively prove the validity of our claims that at the moment should be regarded as hypothetical alternatives to the classical metastatic model.

Although these results are intriguing, still it remains to be established whether the phenotypical changes observed are due to epigenetic or somatic modifications and most importantly whether this phenomenon is applicable to physiological systems in vivo. However, the evidence shown herein that the injection of colon cancer EVs in the tail vein of NOD/SCID mice was associated with malignancy in the lung strengthen the notion that gene transfer by EVs might mediate deregulated growth in target cells and may not be limited to in vitro systems only.

The coexistence of two different cancers in the lungs of the mice injected with cancer EVs seems to support the concept that the phenotype expressed by the transforming cells might have limitations dictated by the nature of the target cell rather than the type of onco-message, as we recently demonstrated in vitro [43].

These results altogether show an alternative mechanism that cancer EVs might implement to modulate the metastatic process. While we are aware that these results do not confirm our belief that metastatic cells might not be circulating cells, in our opinion, they give legitimacy to a model that deserves to be studied more in depth.

We believe that these data add useful pieces of information to the evidence linking circulating cancer-EVs to metastatic risk and clinical outcomes and may guide the development of new and more efficient therapeutic strategies that can target cancer EVs either by inhibiting their production or by blocking their transfer to putative target cells. In addition, the identification of molecular targets whose over-expression is induced by genetic factors derived from EVs, may suggest new therapeutic strategies aimed at inhibiting the metastatic process in its earliest stages.

## Conclusions

The results reported in this work for the first time unveil some of the molecular mechanisms behind the horizontal transfer of malignant traits and confirm the notion that metastatic disease in vitro might be reproduced through transfer of circulating genetic material contained in cancer EVs. We demonstrated that the phenotypical transformation of the fibroblasts was associated with EV-mediated transfer of cancer DNA, coding and non-coding RNA and we showed data that suggest potential transcription of mutated cancer genes in the transformed fibroblasts. The uptake of this genetic material is associated with deregulated expression of transcripts that prompt a mesenchymal to epithelial transition of the fibroblasts that acquire epithelial features and immunohistochemistry markers, suggestive of complete colorectal cancer differentiation. Antagonizing cancer EV uptake could be an effective strategy to block cancer metastasis and should be actively investigated and integrated in metastatic cancer research trials.

## Additional file

Additional file 1: Supplementary materials. (DOC 2447 kb)

## Data Availability

All data generated and analyzed during this study are included in this manuscript and in its supplementary information files.

## Abbreviations

7-AAD: 7-aminoactinomycin D
AO: Acridine orange
BRCA1: Breast cancer 1
BrUTP: Bromouridine triphosphate
CDH1: Cadherin 1; E-cadherin
CTL: Control
DAPI: 4′,6-diamidino-2-phenylindole
DNA: desoxyribonucleic acid
EMT: Epithelial–mesenchymal transition
EVs: Extracellular vesicles
FITC: Fluorescein isothiocyanate
H&E: Hematoxylin and eosin staining
HBSS: Hank’s Balanced Salt Solution
KO: Knock out
MET: Mesenchymal to epithelial transition
miRNA: microRNA
mRNA: messenger RNA
NOD-SCID: nonobese diabetic-severe combined immunodeficiency
PBS: phosphate buffered saline
qPCR: quantitative real-time PCR
RNA: Ribonucleic acid
SNP: Single nucleotide polymorphism
SNV: Single nucleotide variation
