# Supplementary material for: Colorectal cancer-derived extracellular vesicles induce transformation of fibroblasts into colon carcinoma cells
Source: J Exp Clin Cancer Res. 2019 Jun 14;38:257. doi: 10.1186/s13046-019-1248-2 (PMC6567673; doi:10.1186/s13046-019-1248-2)
Supplement: Supplementary file 1 — Supplementary materials. (DOC 2447 kb) [file 13046_2019_1248_MOESM1_ESM.doc]

Colorectal cancer-derived extracellular vesicles induce transformation of fibroblasts into colon carcinoma cells

Mohamed Abdouh^1^, Matteo Floris^2^, Zu-Hua Gao^3^, Vincenzo Arena^4^, Manuel Arena^5^, & Goffredo Orazio Arena^1,6*^

^1^ Cancer Research Program, McGill University Health Centre-Research Institute, 1001 Decarie Boulevard, Montreal, Quebec, Canada, H4A 3J1

^2^ [Department of Biomedical Sciences, Sassari University, Piazza Universita 11, Sassari, Italy](https://www.researchgate.net/institution/Universita_degli_Studi_di_Sassari)

^3^ Department of Pathology, McGill University Health Centre-Research Institute, 1001 Decarie Boulevard, Montreal, Quebec, Canada, H4A 3J1

^4^ Department of Obstetrics and Gynecology, Santo Bambino Hospital, via Torre del Vescovo 4, Catania, Italy

^5^ Department of Surgical Sciences, Organ Transplantation and Advances Technologies, University of Catania, via Santa Sofia 84, Catania, Italy

^6^ Department of Surgery, McGill University, St. Mary Hospital, 3830 Lacombe Avenue, Montreal, Quebec, Canada, H3T 1M5

**Supplementary Material and Methods**

**Scratch assay**

Cells were treated as stated before with control serum EVs and cancer serum EVs. Aliquots were harvested after 1 week, 2 weeks and 3 weeks to perform the scratch assay. Briefly, treated cells were seeded on poly-L-lysine-coated dishes. Cells were plated in medium containing 1% FBS to minimize cell proliferation without affecting cell viability. Cell monolayer was scraped in a straight line to create a “scratch” with a p200 pipet tip and a ruler. Images at the scraped area were captured under a phase-contrast microscope following the scratch and 24 hrs later. Image were analyzed using ImageJ software, and the migration rate was assessed using the following formula:


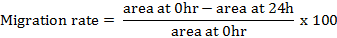


### Immunofluorescence staining

For immunocytofluorometry, cells were fixed in 4% paraformaldehyde (w/v) for 15 min, and permeabilized in PBS/0.3% Triton X-100 for another 15 min. Cells were washed with PBS, and blocked in 2% BSA for 1 h. Primary antibodies against CDX2, CK20, CEA, AE1/AE3 and Vimentin (see Additional File: Table S2) were added to cells at 1:250 dilution in 2% BSA and incubated overnight at 4°C. Cells were washed with PBS and labeled for 1 h with fluorophore-conjugated secondary antibodies (1:500 dilution). After a final wash, the slides were mounted on coverslips with DAPI-containing mounting medium (Vector Laboratories). Cells were visualized using an LSM780 confocal microscope.


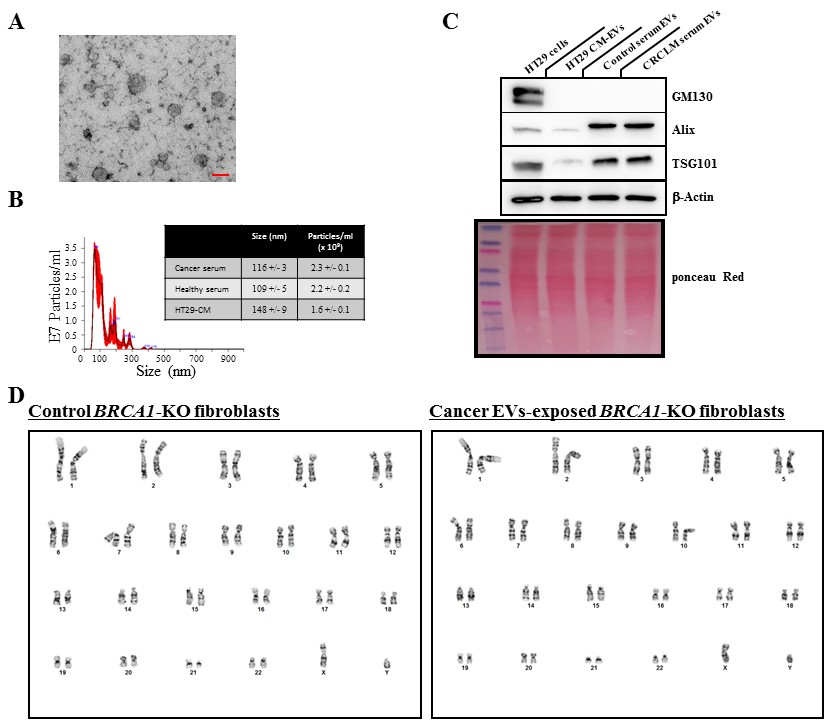


**Supplementary Figure S1. EVs characterization (A-C) and Cells Karyotyping (D).** (A) Representative micrograph of transmission electron microscopy on CRCLM patient sera EV preparations. The image showed small vesicles of approximately 50-120 nm in diameter. Scale bars 100 nm. (B) Nanosight EVs size analyses. Data are expressed as concentration average (black line) +/- standard error (red lines) of six measures and are representative of three EV preparations. (C) Proteins isolated from HT29 cells, or EVs from HT29 cells-derived conditioned medium (CM), or healthy patient serum, or CRCRLM patient serum were analyzed by Western blot for the expression of specific markers. β-actin and ponceau red membrane labeling were used for the control of proteins loading. (D) Karyotype analyses of control EVs-exposed and cancer EVs-exposed *BRCA1*-KO fibroblasts. Colon cancer EVs were isolated from the serum of a female colon cancer patients. Standard G-banding revealed a normal male karyotype as 46,XY in both samples.


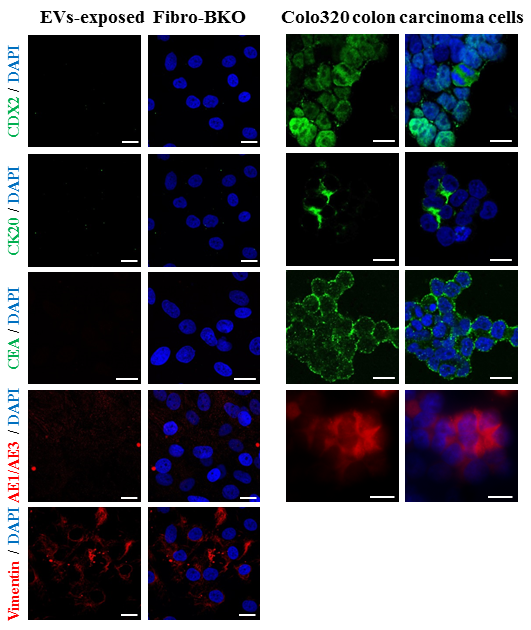


**Supplementary Figure S2. *BRCA1*-KO fibroblasts exposed to cancer EVs failed to change their fate during *in vitro* culture.** *BRCA1*-KO fibroblasts were treated for 3 weeks with EVs isolated from cancer patient sera. Cells were plated on chamber slides and processed for immunofluorescence using antibodies to CDX2, CK20, CEA, AE1/AE2 and Vimentin. Labeled cells were counterstained with DAPI for nuclei visualization. Colo320 colon carcinoma cell line was used as positive control for antibodies performance. Scale bars; 15 µm.


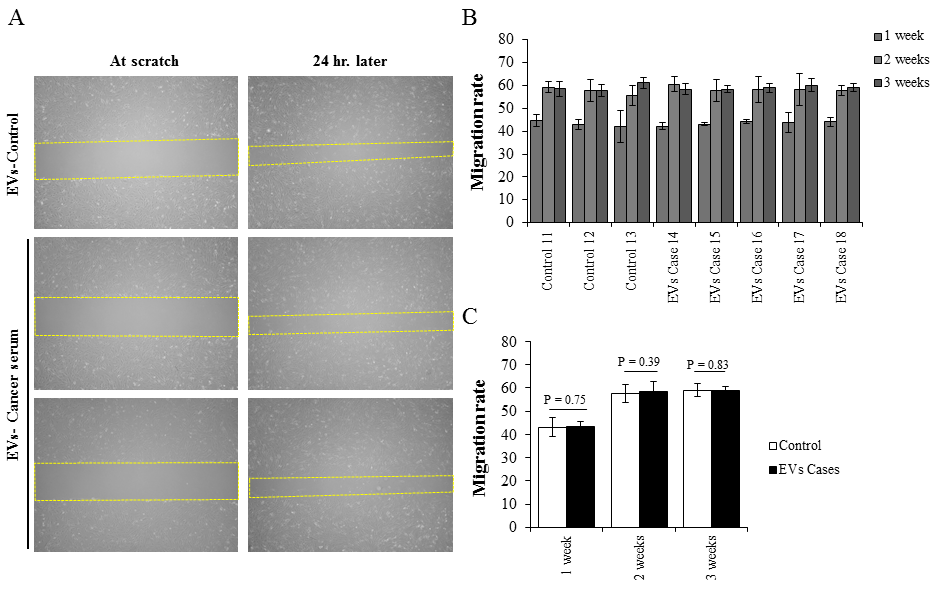


**Supplementary Figure S3. *BRCA1*-KO fibroblasts exposed to cancer EVs failed to change their migratory faculty.** *BRCA1*-KO fibroblasts were treated for 1, 2 or 3 weeks with EVs isolated from control sera (n = 3) or cancer patient sera (n = 5). (A) Cell monolayers were scraped at confluence, and cell migration was assessed 24 hr later. Areas of migration were measured in triplicates for each sample. (B and C) Migration rate were represented as mean ± SD for each sample (B), and after pooling data for control serum EVs and cancer serum EVs (C), respectively.


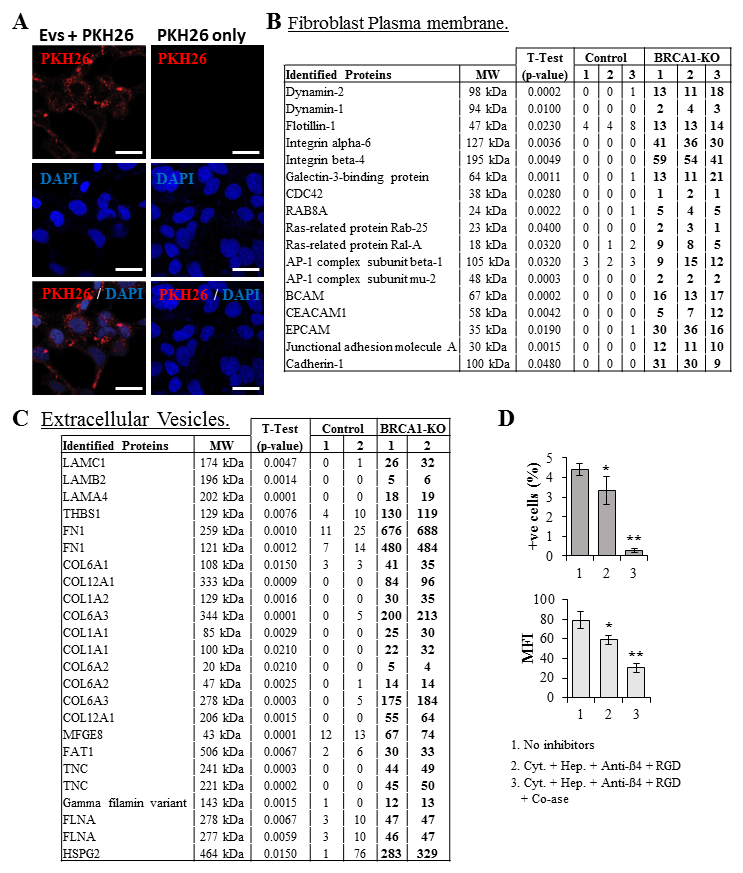


**Supplementary Figure S4. *BRCA1*-KO fibroblasts actively uptake cancer EVs**. **A,** Representative images of confocal microscopy monitoring PKH-26-labeled EVs (red dots) uptake *in vitro* into *BRCA1*-KO fibroblasts. EVs were internalized efficiently, dispersed in the cytoplasm and tended to form aggregates in the perinuclear regions. Nuclei were colored with DAPI (blue). Scale bars: 20 µm. **B,** Plasma membrane proteins involved in EVs uptake and that are overexpressed in *BRCA1*-KO fibroblasts. n = 3 plasma membrane preparations. **C,** EVs proteins involved in EVs uptake that are overexpressed in BRCA1-KO fibroblasts-derived EVs. *BRCA1*-KO and control fibroblasts were treated for 3 weeks with serum from patients with CRC-LM, or serum from healthy donor, respectively. Following 3 weeks treatment, cells were maintained in EV-free FBS to collect conditioned media. EVs were isolated from the respective conditioned media and subjected to mass spectrometry analyses. n = 2 EVs preparations. **D,** EVs were isolated and labeled with PKH-26. Cells were treated or not with Cytostatin (Cyt.: 1.4 μg/ml), Heparin (Hep.: 10 μg/ml) and the anti-β4 integrin antibody (Anti-β4: 10 μg/ml). In parallel, EVs were treated or not with RGD (300 nM) and Collagenase I (Co-ase: 500 μg/ml). Cells were exposed to EVs and analyzed by flow cytometry. Data are expressed as the percentage of PKH-26 positive cells (top panel) and mean fluorescence intensity (MFI; bottom panel). Data are mean +/- SD. n = 3 independent experiments. *P < 0.05, **P < 0.01.


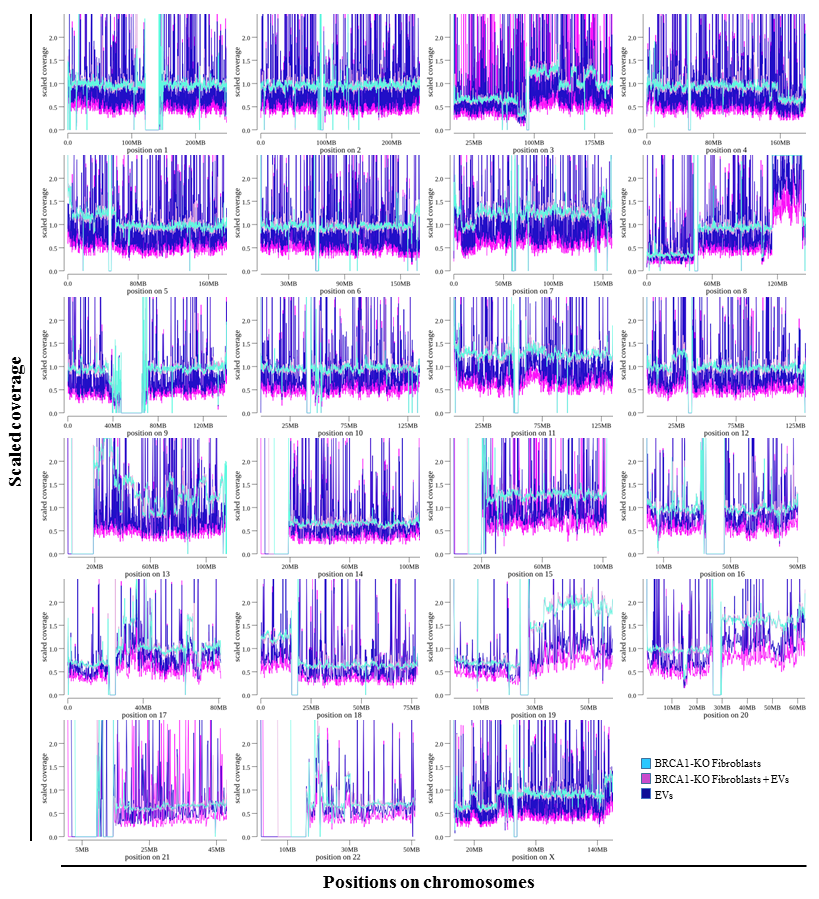


**Supplementary Figure S5.** Chromosomal coverage of analyzed samples.


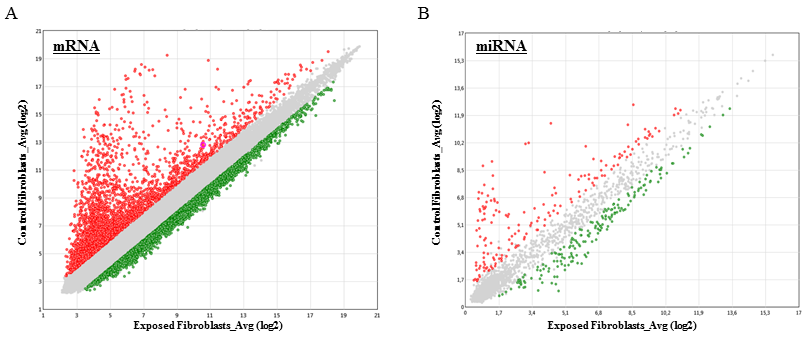


**Supplementary Figure S6.** Scatter plot of (A) mRNA and (B) miRNA expression profiles of *BRCA1*-KO fibroblasts prior and following exposure to cancer EVs. Green dots are transcripts overexpressed in EVs-exposed cells. Red dots are transcripts downregulated in EVs-exposed cells. Data were obtained with 3 independent cell cultures. Filter criteria were set as follows: Fold change: > 2 or < -2 and FDR P value < 0,05.


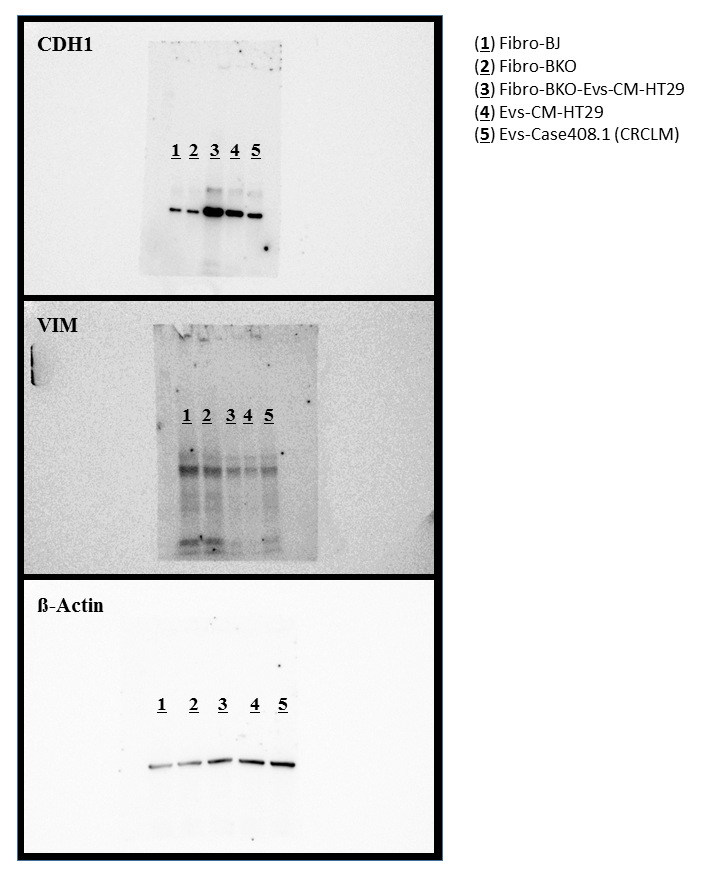


**Supplementary Figure S7.** Whole blots representing data shown in Figure 6B.

**Supplementary Table S1. Primer sequences.**

|  | Forward primers | Reverse primers |
| --- | --- | --- |
| GAPDH | TGACAACTTTGGTATCGTGGAAGG | AGGGATGATGTTCTGGAGAGCC |
| ZEB1 | AAGAACTGCTGGGAGGATGA | TCTGCATCTGACTCGCATTC |
| ZEB2 | CGCTTGACATCACTGAAGGA | GCTCCTTGGGTTAGCATTTG |
| SNAI1 | CCCAATCGGAAGCCTAACTA | ACAGAGTCCCAGATGAGCATT |
| SNAI2 | CCTTCCTGGTCAAGAAGCAT | TTGTGGTATGACAGGCATGG |
| CDH1 | CTGGGTTATTCCTCCCATCA | CACCTTCAGCCATCCTGTTT |
| CDH2 | AGGGGACCTTTTCCTCAAGA | CCGAGATGGGGTTGATAATG |
| Vimentin | TGTCCAAATCGATGTGGATGTTTC | TTGTACCATTCTTCTGCCTCCTG |
| Fibronectin | CCACCCCCATAAGGCATAGG | GTAGGGGTCAAAGCACGAGTCATC |
| CDKN1A | AGGGGACAGCAGAGGAAG | GCGTTTGGAGTGGTAGAAATCTG |
| MYC | CTTCTCTCCGTCCTCGGATTCT | GAAGGTGATCCAGACTCTGACCTT |
| HRAS | TACGGCATCCCCTACATCGAGAC | CACCAACGTGTAGAAGGCATCCTC |
| MDM2 | GCAGTGAATCTACAGGGACGC | ATCCTGATCCAACCAATCACC |
| BCL2L1 | ATGGCAGCAGTAAAGCAAGC | CGGAAGAGTTCATTCACTACCTGT |

**Supplementary Table S**2. List of antibodies used in this study.

| **Antibodies ID** | **Species** | **Manufacturer** |
| --- | --- | --- |
| Ki67 | Rabbit Monoclonal | Ventana (USA) |
| AE1/AE3 | Mouse Monoclonal | DAKO (Denmark) |
| CK7 | Mouse Monoclonal | DAKO (Denmark) |
| CK20 | Mouse Monoclonal | DAKO (Denmark) |
| CDX-2 | Rabbit Monoclonal | Cell MARQUE (USA) |
| CDH1 | Rabbit Monoclonal | DAKO (Denmark) |
| Vimentin | Mouse Monoclonal | Ventana (USA) |
| CEA-P | Rabbit Polyclonal | DAKO (Denmark) |
| Napsin | Mouse Monoclonal | Ventana (USA) |
| TTF1 | Mouse Monoclonal | Ventana (USA) |

**Supplementary Table S3**

| **Chr** | **Position** | **Ref.** | **Alt.** | **Function** | **Gene ID** | **AA Change** | **Validated** |
| --- | --- | --- | --- | --- | --- | --- | --- |
| 1 | 53792651 | A | C | exonic | LRP8 | D46E | **yes** |
| 1 | 148252777 | T | TCTC | exonic | NBPF14 | UNKNOWN | **yes** |
| 1 | 197070442 | G | T | exonic | ASPM | L2647I | **yes** |
| 2 | 48602252 | C | T | exonic | FOXN2 | S322S | **yes** |
| 3 | 49726070 | G | A | exonic | MST1 | P19S | **yes** |
| 3 | 49832788 | G | A | exonic | CDHR4 | T291M | **yes** |
| 3 | 51422766 | G | T | exonic | MANF | R1M | **yes** |
| 4 | 367267 | T | C | exonic | ZNF141 | A347A | **yes** |
| 4 | 367275 | A | G | exonic | ZNF141 | Q350R | **yes** |
| 4 | 164085425 | T | C | exonic | NAF1 | I162V | **yes** |
| 6 | 32489925 | C | T | exonic | HLA-DRB5 | E43K | **yes** |
| 6 | 32489939 | T | C | exonic | HLA-DRB5 | Q38R | **yes** |
| 6 | 32489943 | A | G | exonic | HLA-DRB5 | L37L | **yes** |
| 6 | 32713070 | A | C | exonic | HLA-DQA2 | M73L | **yes** |
| 6 | 32713075 | T | C | exonic | HLA-DQA2 | F74F | **yes** |
| 6 | 32713076 | A | C | exonic | HLA-DQA2 | S75R | **yes** |
| 6 | 32713080 | A | G | exonic | HLA-DQA2 | K76R | **yes** |
| 6 | 32713086 | T | G | exonic | HLA-DQA2 | I78R | **yes** |
| 6 | 32713090 | T | A | exonic | HLA-DQA2 | S79R | **yes** |
| 6 | 32713102 | G | A | exonic | HLA-DQA2 | Q83Q | **yes** |
| 6 | 32713103 | A | T | exonic | HLA-DQA2 | S84C | **yes** |
| 6 | 32713104 | G | T | exonic | HLA-DQA2 | S84I | **yes** |
| 6 | 32713113 | G | C | exonic | HLA-DQA2 | R87T | **yes** |
| 6 | 32713117 | T | C | exonic | HLA-DQA2 | N88N | **yes** |
| 6 | 32713120 | G | C | exonic | HLA-DQA2 | M89I | **yes** |
| 6 | 32713127 | G | C | exonic | HLA-DQA2 | G92R | **yes** |
| 6 | 32713128 | G | T | exonic | HLA-DQA2 | G92V | **yes** |
| 6 | 37623566 | C | T | exonic | MDGA1 | P163P | **yes** |
| 7 | 150888788 | A | G | exonic | IQCA1L | UNKNOWN | **yes** |
| 9 | 135946015 | T | C | exonic | CEL | I488T | **yes** |
| 9 | 139250186 | C | T | exonic | GPSM1 | D500D | **yes** |
| 11 | 64083290 | G | A | exonic | ESRRA | R375Q | **yes** |
| 11 | 64083293 | G | T | exonic | ESRRA | R376L | **yes** |
| 11 | 64083300 | G | A | exonic | ESRRA | A378A | **yes** |
| 11 | 64083320 | T | C | exonic | ESRRA | L385P | **yes** |
| 11 | 120175749 | A | G | exonic | POU2F3 | H152R | **yes** |
| 11 | 121038810 | C | T | exonic | TECTA | S1878S | **yes** |
| 12 | 319111 | T | C | exonic | SLC6A12 | A14A | **yes** |
| 12 | 6711147 | C | A | exonic | CHD4 | E139D | **yes** |
| 12 | 42499817 | A | ATT | exonic | GXYLT1 | UNKNOWN | **yes** |
| 14 | 88652389 | C | T | exonic | KCNK10 | A374A | **yes** |
| 14 | 105412005 | A | G | exonic | AHNAK2 | D3261D | **yes** |
| 14 | 105412009 | A | G | exonic | AHNAK2 | M3260T | **yes** |
| 15 | 71125204 | A | G | exonic | LARP6 | F221F | **yes** |
| 16 | 1291928 | C | T | exonic | TPSAB1 | D200D | **yes** |
| 16 | 20043330 | T | C | exonic | GPR139 | V170V | **yes** |
| 16 | 55866949 | T | C | exonic | CES1 | I7V | **yes** |
| 16 | 55866952 | A | G | exonic | CES1 | F6L | **yes** |
| 16 | 55866953 | G | A | exonic | CES1 | A5A | **yes** |
| 16 | 55866957 | C | G | exonic | CES1 | R4P | **yes** |
| 17 | 39197618 | A | T | exonic | KRTAP1-1 | F11Y | **yes** |
| 17 | 39296412 | T | A | exonic | KRTAP4-6 | S110C | **yes** |
| 17 | 39296422 | A | G | exonic | KRTAP4-6 | R106R | **yes** |
| 17 | 39383027 | G | T | exonic | KRTAP9-2 | A41S | **yes** |
| 19 | 8999517 | T | A | exonic | MUC16 | Y13553F | **yes** |
| 19 | 54754838 | T | TG | exonic | LILRB5 | UNKNOWN | **yes** |
| 19 | 56029552 | G | GACTG | exonic | SSC5D | UNKNOWN | **yes** |
| 22 | 42523505 | C | T | exonic | CYP2D6 | G373S | **yes** |

**Supplementary Table S4. *BRCA1*-mutated fibroblasts exposed to cancer cells EVs undergo Mesenchymal to Epithalial Transition (MET), increased proliferation and reduced apoptosis. Data were obtained with 3 independent cell cultures. Filter criteria were set as follows: Fold change > 2 (downexpressed in EVs-exposed cells) or < -2 (overexpressed in EVs-exposed cells) and FDR P value < 0,05.**

|  | **Up-regulated in EV-exposed cells** | | | **Down-regulated in EV-exposed cells** | | |
| --- | --- | --- | --- | --- | --- | --- |
| **Pathways** | **Gene Symbol** | **Fold Change** | **FDR P-val** | **Gene Symbol** | **Fold Change** | **FDR P-val** |
| **MET** | PROX1 | -3,40 | 7,62E-06 | FN1 | 3460,19 | 4,91E-10 |
|  | CLDN2 | -3,23 | 0,0002 | MMP2 | 3057,82 | 2,83E-08 |
|  | MMP15 | -3,21 | 4,34E-05 | SPARC | 2617,74 | 2,18E-06 |
|  | CLDN4 | -3,02 | 4,38E-06 | SNAI2 | 2113,57 | 2,22E-12 |
|  | CLDN7 | -2,99 | 3,76E-06 | PRRX1 | 1153,59 | 6,94E-11 |
|  | OCLN | -2,86 | 5,60E-06 | ITGA5 | 399,52 | 1,66E-10 |
|  | JUP | -2,85 | 4,12E-05 | CDH2 | 230,38 | 4,90E-09 |
|  | TMPRSS4 | -2,84 | 2,65E-05 | TWIST2 | 215,30 | 3,87E-10 |
|  | PKP2 | -2,82 | 6,69E-05 | ZEB1 | 66,26 | 9,50E-09 |
|  | CDH1 | -2,71 | 0,0004 | SPOCK1 | 58,05 | 3,03E-09 |
|  | CLDN3 | -2,66 | 7,19E-05 | COL4A1 | 17,75 | 1,87E-08 |
|  | CLDN1 | -2,66 | 4,03E-05 | SNAI1 | 12,98 | 2,51E-07 |
|  | GDF15 | -2,61 | 4,14E-05 | CLDN11 | 12,96 | 4,92E-09 |
|  | MAP2K6 | -2,56 | 0,0013 | ID2 | 10,40 | 1,87E-08 |
|  | SUZ12 | -2,28 | 0,0001 | ZEB2 | 9,84 | 1,60E-07 |
|  | PKD1P5 | -2,13 | 8,41E-05 | SMAD4 | 5,39 | 3,73E-06 |
|  | EZH2 | -2,12 | 9,27E-05 | TGFB1 | 4,29 | 1,26E-06 |
|  | PKD1 | -2,04 | 0,0015 | COL4A2 | 4,18 | 6,67E-06 |
|  | HRAS | -2,01 | 7,74E-05 | HIF1A | 3,37 | 1,13E-05 |
|  | MAP2K1 | -2,01 | 0,0004 | CDKL2 | 2,59 | 0,0216 |
|  |  |  |  | NRP2 | 2,59 | 0,0048 |
|  |  |  |  | SHC1 | 2,34 | 0,0001 |
|  |  |  |  | TWIST1 | 2,17 | 0,002 |
| **Cell cycle progression** | MYC | -2,74 | 1,51E-05 | AKT3 | 25,01 | 4,49E-09 |
|  | PIK3CB | -2,29 | 0,0105 | CDKN1A | 16,95 | 1,09E-07 |
|  | HRAS | -2,01 | 7,74E-05 | SHC1 | 2,34 | 0,0001 |
| **Cell Survival** | MYB | -2,89 | 2,70E-05 | AKT3 | 25,01 | 4,49E-09 |
|  | BCL2L1 | -2,31 | 5,54E-05 | MDM2 | 2,97 | 2,32E-05 |
|  | PIK3CB | -2,29 | 0,0105 | SHC1 | 2,34 | 0,0001 |
|  | HRAS | -2,01 | 7,74E-05 |  |  |  |

**Supplementary Table S5.** mRNA / miRNA interaction network derived from the microarray analyses depicting the transcripts involved in MET process. Connections were retrieved from public database. Data were obtained with 3 independent cell cultures. Filter criteria were set as follows: Fold change > 2 (downexpressed in EVs-exposed cells) or < -2 (overexpressed in EVs-exposed cells) and FDR P value < 0,05.

| **miRNA overexpressed in exposed cells** | | | |  |  |  |  |  |
| --- | --- | --- | --- | --- | --- | --- | --- | --- |
| **Target** | **Transcript ID** | **Fold Change** | **FDR P-val** |  | **Target** | **Transcript ID** | **Fold Change** | **FDR P-val** |
| **ZEB1** | hsa-miR-203a | -8,17 | 0,0002 |  | **AKT3** | hsa-miR-15a-5p | -2,89 | 0,0019 |
|  | hsa-miR-141-3p | -6,39 | 0,0004 |  |  | hsa-miR-7854-3p | -2,82 | 0,0013 |
|  | hsa-miR-183-5p | -4,31 | 0,0002 |  |  | hsa-miR-361-5p | -2,6 | 8,22E-06 |
|  | hsa-miR-200b-3p | -4,1 | 0,0003 |  |  | hsa-miR-16-5p | -2,48 | 7,99E-06 |
|  | hsa-miR-200a-3p | -3,72 | 9,34E-05 |  | **TGFB1** | hsa-miR-17-5p | -3,41 | 4,54E-07 |
|  | hsa-miR-130b-3p | -3,39 | 1,12E-06 |  |  | hsa-miR-93-5p | -2,68 | 8,02E-06 |
|  | hsa-miR-629-5p | -3,35 | 8,86E-05 |  |  | hsa-miR-19b-3p | -2,12 | 0,002 |
|  | hsa-miR-205-5p | -2,98 | 0,0004 |  |  | hsa-miR-324-3p | -2,06 | 4,13E-05 |
|  | hsa-miR-150-5p | -2,73 | 0,0002 |  | **HIF1A** | hsa-miR-1910-3p | -4,54 | 0,0004 |
|  | hsa-miR-200c-3p | -2,21 | 5,92E-06 |  |  | hsa-miR-18a-5p | -4,39 | 2,11E-06 |
| **ZEB2** | hsa-miR-203a | -8,17 | 0,0002 |  |  | hsa-miR-210-3p | -4,19 | 1,51E-07 |
|  | hsa-miR-141-3p | -6,39 | 0,0004 |  |  | hsa-miR-20a-5p | -3,83 | 2,08E-07 |
|  | hsa-miR-200b-3p | -4,1 | 0,0003 |  |  | hsa-miR-106b-5p | -3,83 | 1,16E-06 |
|  | hsa-miR-200a-3p | -3,72 | 9,34E-05 |  |  | hsa-miR-17-5p | -3,41 | 4,54E-07 |
|  | hsa-miR-205-5p | -2,98 | 0,0004 |  |  | hsa-miR-20b-5p | -3,26 | 0,0001 |
|  | hsa-miR-16-5p | -2,48 | 7,99E-06 |  |  | hsa-miR-151a-3p | -3 | 6,46E-07 |
|  | hsa-miR-200c-3p | -2,21 | 5,92E-06 |  |  | hsa-miR-18b-5p | -2,71 | 0,005 |
| **SNAI1** | hsa-miR-203a | -8,17 | 0,0002 |  |  | hsa-miR-93-5p | -2,68 | 8,02E-06 |
|  | hsa-miR-30d-5p | -3,15 | 4,19E-06 |  |  | hsa-miR-107 | -2,43 | 4,41E-06 |
|  | hsa-miR-30b-5p | -2,63 | 0,0001 |  |  | hsa-miR-106b-3p | -2,03 | 6,31E-05 |
| **SNAI2** | hsa-miR-203a | -8,17 | 0,0002 |  | **SMAD4** | hsa-miR-203a | -8,17 | 0,0002 |
|  | hsa-miR-183-5p | -4,31 | 0,0002 |  |  | hsa-miR-452-5p | -7,44 | 2,04E-07 |
|  | hsa-miR-182-5p | -4,23 | 7,58E-07 |  |  | hsa-miR-18a-5p | -4,39 | 2,11E-06 |
|  | hsa-miR-128-3p | -4,08 | 3,48E-06 |  |  | hsa-miR-183-5p | -4,31 | 0,0002 |
|  | hsa-miR-148b-3p | -2,13 | 0,0139 |  |  | hsa-miR-182-5p | -4,23 | 7,58E-07 |
| **TWIST1** | hsa-miR-106b-5p | -3,83 | 1,16E-06 |  |  | hsa-miR-20a-5p | -3,83 | 2,08E-07 |
|  | hsa-miR-361-5p | -2,6 | 8,22E-06 |  |  | hsa-miR-106b-5p | -3,83 | 1,16E-06 |
| **Vimentin** | hsa-miR-17-3p | -13,32 | 1,36E-05 |  |  | hsa-miR-224-5p | -3,69 | 0,0003 |
|  | hsa-miR-1287-5p | -3,72 | 3,61E-05 |  |  | hsa-miR-106a-5p | -3,53 | 5,32E-07 |
|  | hsa-miR-17-5p | -3,41 | 4,54E-07 |  |  | hsa-miR-421 | -3,52 | 0,0002 |
|  | hsa-miR-378a-3p | -3,1 | 5,87E-06 |  |  | hsa-miR-17-5p | -3,41 | 4,54E-07 |
|  | hsa-miR-16-5p | -2,48 | 7,99E-06 |  |  | hsa-miR-130b-3p | -3,39 | 1,12E-06 |
|  | hsa-miR-615-3p | -2,16 | 4,11E-05 |  |  | hsa-miR-20b-5p | -3,26 | 0,0001 |
| **CDH2** | hsa-miR-27b-5p | -6,06 | 0,0002 |  |  | hsa-miR-205-5p | -2,98 | 0,0004 |
|  | hsa-miR-194-5p | -3,14 | 6,71E-07 |  |  | hsa-miR-93-5p | -2,68 | 8,02E-06 |
|  | hsa-miR-615-3p | -2,16 | 4,11E-05 |  |  | hsa-miR-19b-3p | -2,12 | 0,002 |
| **SHC1** | hsa-miR-141-3p | -6,39 | 0,0004 |  | **MMP2** | hsa-miR-452-5p | -7,44 | 2,04E-07 |
|  | hsa-miR-200b-3p | -4,1 | 0,0003 |  |  | hsa-miR-29b-2-5p | -6,12 | 0,0001 |
|  | hsa-miR-200a-3p | -3,72 | 9,34E-05 |  |  | hsa-miR-106b-5p | -3,83 | 1,16E-06 |
|  | hsa-miR-30b-5p | -2,63 | 0,0001 |  |  | hsa-miR-491-5p | -3,5 | 0,0022 |
|  | hsa-miR-200c-3p | -2,21 | 5,92E-06 |  |  | hsa-miR-17-5p | -3,41 | 4,54E-07 |
| **NRP2** | hsa-miR-196a-5p | -4,66 | 0,0012 |  |  | hsa-miR-708-5p | 2,48 | 0,0084 |
|  | hsa-miR-331-3p | -4,03 | 0,0008 |  | **COL4A1** | hsa-miR-421 | -3,52 | 0,0002 |
| **CDKL2** | hsa-miR-203a | -8,17 | 0,0002 |  |  | hsa-miR-769-3p | -3,15 | 6,29E-05 |
| **ITGA5** | hsa-miR-183-5p | -4,31 | 0,0002 |  |  | hsa-miR-16-5p | -2,48 | 7,99E-06 |
|  | hsa-miR-205-5p | -2,98 | 0,0004 |  |  | hsa-miR-18a-3p | -2,19 | 0,0002 |
|  | hsa-miR-1229-3p | -2,87 | 0,0001 |  | **COL4A2** | hsa-miR-210-3p | -4,19 | 1,51E-07 |
|  | hsa-miR-31-5p | -2,28 | 3,97E-05 |  |  | hsa-miR-10a-5p | -4,05 | 0,0001 |
|  | hsa-miR-484 | -2,17 | 0,0065 |  |  | hsa-miR-16-5p | -2,48 | 7,99E-06 |
|  | hsa-miR-148b-3p | -2,13 | 0,0139 |  |  | hsa-miR-615-3p | -2,16 | 4,11E-05 |
| **SPARC** | hsa-miR-10a-5p | -4,05 | 0,0001 |  | **MDM2** | hsa-miR-192-3p | -7,78 | 6,53E-06 |
|  | hsa-miR-192-5p | -2,98 | 1,20E-06 |  |  | hsa-miR-500a-3p | -5,82 | 3,85E-05 |
|  | hsa-miR-432-5p | 239,51 | 2,32E-07 |  |  | hsa-miR-224-3p | -5,25 | 0,0001 |
| **ID2** | hsa-miR-378a-3p | -3,1 | 5,87E-06 |  |  | hsa-miR-200c-5p | -4,09 | 9,37E-06 |
|  | hsa-miR-192-5p | -2,98 | 1,20E-06 |  |  | hsa-miR-20a-5p | -3,83 | 2,08E-07 |
|  | hsa-miR-103a-3p | -2,39 | 6,27E-06 |  |  | hsa-miR-106b-5p | -3,83 | 1,16E-06 |
|  | hsa-miR-199a-3p | 88,17 | 9,89E-08 |  |  | hsa-miR-185-5p | -3,8 | 5,65E-07 |
|  | hsa-miR-199b-3p | 88,17 | 9,89E-08 |  |  | hsa-miR-550a-3p | -3,78 | 0,0001 |
|  | hsa-miR-125b-5p | 133,82 | 1,23E-05 |  |  | hsa-miR-330-3p | -3,54 | 3,57E-05 |
| **CDKN1A** | hsa-miR-182-5p | -4,23 | 7,58E-07 |  |  | hsa-miR-106a-5p | -3,53 | 5,32E-07 |
|  | hsa-miR-20a-5p | -3,83 | 2,08E-07 |  |  | hsa-miR-4306 | -3,42 | 0,0228 |
|  | hsa-miR-106b-5p | -3,83 | 1,16E-06 |  |  | hsa-miR-17-5p | -3,41 | 4,54E-07 |
|  | hsa-miR-106a-5p | -3,53 | 5,32E-07 |  |  | hsa-miR-6516-5p | -3,32 | 0,006 |
|  | hsa-miR-17-5p | -3,41 | 4,54E-07 |  |  | hsa-miR-20b-5p | -3,26 | 0,0001 |
|  | hsa-miR-20b-5p | -3,26 | 0,0001 |  |  | hsa-miR-25-3p | -2,86 | 6,95E-05 |
|  | hsa-miR-15a-5p | -2,89 | 0,0019 |  |  | hsa-miR-425-5p | -2,8 | 8,34E-06 |
|  | hsa-miR-1229-3p | -2,87 | 0,0001 |  |  | hsa-miR-18b-5p | -2,71 | 0,005 |
|  | hsa-miR-345-5p | -2,76 | 0,0013 |  |  | hsa-miR-93-5p | -2,68 | 8,02E-06 |
|  | hsa-miR-93-5p | -2,68 | 8,02E-06 |  |  | hsa-miR-664b-3p | -2,3 | 0,0473 |
|  | hsa-miR-28-5p | -2,67 | 0,0003 |  |  | hsa-miR-484 | -2,17 | 0,0065 |
|  | hsa-let-7g-5p | -2,6 | 0,0278 |  | **FN1** | hsa-miR-200b-3p | -4,1 | 0,0003 |
|  | hsa-miR-16-5p | -2,48 | 7,99E-06 |  |  | hsa-let-7g-5p | -2,6 | 0,0278 |
|  | hsa-let-7i-5p | -2,41 | 0,0013 |  |  | hsa-miR-200c-3p | -2,21 | 5,92E-06 |
|  | hsa-miR-18a-3p | -2,19 | 0,0002 |  |  | hsa-miR-615-3p | -2,16 | 4,11E-05 |
|  |  |  |  |  |  |  |  |  |
| **miRNA downexpressed in exposed cells** | | | |  |  |  |  |  |
| **Target** | **Transcript ID** | **Fold Change** | **FDR P-val** |  | **Target** | **Transcript ID** | **Fold Change** | **FDR P-val** |
| **CDH1** | hsa-miR-138-5p | 36,3 | 2,09E-08 |  | **MYC** | hsa-miR-92b-3p | 2,17 | 0,0053 |
|  | hsa-miR-199a-5p | 68,7 | 1,16E-08 |  |  | hsa-miR-6765-5p | 2,3 | 0,0001 |
| **PKP2** | hsa-miR-34a-5p | 5,31 | 8,27E-07 |  |  | hsa-miR-30a-3p | 3,51 | 0,0002 |
| **TMPRSS4** | hsa-miR-543 | 9,62 | 0,0003 |  |  | hsa-miR-155-5p | 4,09 | 6,57E-05 |
| **PKD1** | hsa-miR-6813-5p | 2,07 | 0,0339 |  |  | hsa-miR-34a-5p | 5,31 | 8,27E-07 |
|  | hsa-miR-6726-5p | 2,12 | 0,0006 |  |  | hsa-miR-487b-3p | 26,85 | 4,55E-09 |
|  | hsa-miR-6722-3p | 2,17 | 0,0001 |  |  | hsa-miR-145-5p | 134,23 | 1,58E-06 |
|  | hsa-miR-6132 | 2,26 | 1,98E-05 |  | **JUP** | hsa-miR-155-5p | 4,09 | 6,57E-05 |
|  | hsa-miR-6090 | 2,27 | 0,0032 |  | **HRAS** | hsa-miR-663a | 3,12 | 1,63E-06 |
|  | hsa-miR-4763-3p | 2,34 | 0,0069 |  |  | hsa-miR-143-3p | 29,11 | 3,47E-08 |
|  | hsa-miR-6791-5p | 3,07 | 0,0031 |  | **SUZ12** | hsa-miR-155-5p | 4,09 | 6,57E-05 |
|  | hsa-miR-6789-5p | 4,49 | 3,57E-05 |  |  | hsa-miR-3185 | 7,36 | 1,04E-05 |
|  | hsa-miR-1909-3p | 6,1 | 3,18E-05 |  |  | hsa-miR-487b-3p | 26,85 | 4,55E-09 |
|  | hsa-miR-486-3p | 18,38 | 0,0002 |  |  | hsa-miR-138-5p | 36,3 | 2,09E-08 |
| **MYB** | hsa-miR-155-5p | 4,09 | 6,57E-05 |  | **EZH2** | hsa-miR-92b-3p | 2,17 | 0,0053 |
|  | hsa-miR-34a-5p | 5,31 | 8,27E-07 |  |  | hsa-miR-708-5p | 2,48 | 0,0084 |
| **MAP2K6** | hsa-miR-145-5p | 134,23 | 1,58E-06 |  |  | hsa-miR-138-5p | 36,3 | 2,09E-08 |
| **CLDN1** | hsa-miR-155-5p | 4,09 | 6,57E-05 |  |  | hsa-miR-199a-5p | 68,7 | 1,16E-08 |
| **MAP2K1** | hsa-miR-34a-5p | 5,31 | 8,27E-07 |  |  | hsa-miR-214-3p | 127,29 | 3,26E-06 |
